# Metal Fluorides: Tools for Structural and Computational Analysis of Phosphoryl Transfer Enzymes

**DOI:** 10.1007/s41061-017-0130-y

**Published:** 2017-03-15

**Authors:** Yi Jin, Robert W. Molt, G. Michael Blackburn

**Affiliations:** 1 Structural Biology Laboratory, Department of Chemistry, University of York, York, YO31 7YD UK; 2 Department of Molecular Biology and Biotechnology, Krebs Institute, University of Sheffield, Sheffield, S10 2TN UK; 3 ENSCO, Inc., 4849 North Wickham Road, Melbourne, FD 32940 USA; 4 Department of Chemistry and Chemical Biology, Indiana University-Purdue University, Indianapolis, IN 46202 USA; 5 Department of Biochemistry and Molecular Biology, School of Medicine, Indiana University, Indianapolis, IN 46202 USA

**Keywords:** MF_*x*_, Phosphoryl group surrogates, Enzyme mechanisms, Transition state analogs, QM/MM computation, KS-DFT analysis

## Abstract

The phosphoryl group, PO_3_^–^, is the dynamic structural unit in the biological chemistry of phosphorus. Its transfer from a donor to an acceptor atom, with oxygen much more prevalent than nitrogen, carbon, or sulfur, is at the core of a great majority of enzyme-catalyzed reactions involving phosphate esters, anhydrides, amidates, and phosphorothioates. The serendipitous discovery that the phosphoryl group could be labeled by “nuclear mutation,” by substitution of PO_3_^–^ by MgF_3_^–^ or AlF_4_^–^, has underpinned the application of metal fluoride (MF_*x*_) complexes to mimic transition states for enzymatic phosphoryl transfer reactions, with sufficient stability for experimental analysis. Protein crystallography in the solid state and ^19^F NMR in solution have enabled direct observation of ternary and quaternary protein complexes embracing MF_*x*_ transition state models with precision. These studies have underpinned a radically new mechanistic approach to enzyme catalysis for a huge range of phosphoryl transfer processes, as varied as kinases, phosphatases, phosphomutases, and phosphohydrolases. The results, without exception, have endorsed trigonal bipyramidal geometry (tbp) for concerted, “in-line” stereochemistry of phosphoryl transfer. QM computations have established the validity of tbp MF_*x*_ complexes as reliable models for true transition states, delivering similar bond lengths, coordination to essential metal ions, and virtually identical hydrogen bond networks. The emergence of protein control of reactant orbital overlap between bond-forming species within enzyme transition states is a new challenging theme for wider exploration.

## Background

Alexander Todd^1^ and Frank Westheimer^2^ held complementary, and sometime overlapping, views on the centrality of phosphates for life. Todd’s pronouncement: “Where there’s Life, there’s Phosphorus”, encapsulated his conviction that enzymes that manipulate phosphates have been at the heart of biology from the dawn of life anywhere in the universe [1]. Westheimer identified the evolutionary centrality of phosphate [2]. The cellular behavior of phosphate esters and anhydrides provides one of the most remarkable chemical paradoxes: phosphate monoesters hydrolyze spontaneously under physiological conditions with *t*_1/2_ 10^12^ years, yet simple phosphatase enzymes have *k*_cat_ ca. 30 s^−1^. The enormous difference corresponds to a remarkable catalytic rate enhancement of 10^21^ [3]. How do enzymes achieve this? This article focuses on the use of aluminum and magnesium fluoride complexes to mimic structures of transition states of enzymatic reactions that involve the phosphoryl group, PO_3_^−^, and to provide a structural base for quantum chemical computations to describe them in detail.

### Basics of Phosphoryl Transfer

**Scheme 1:**
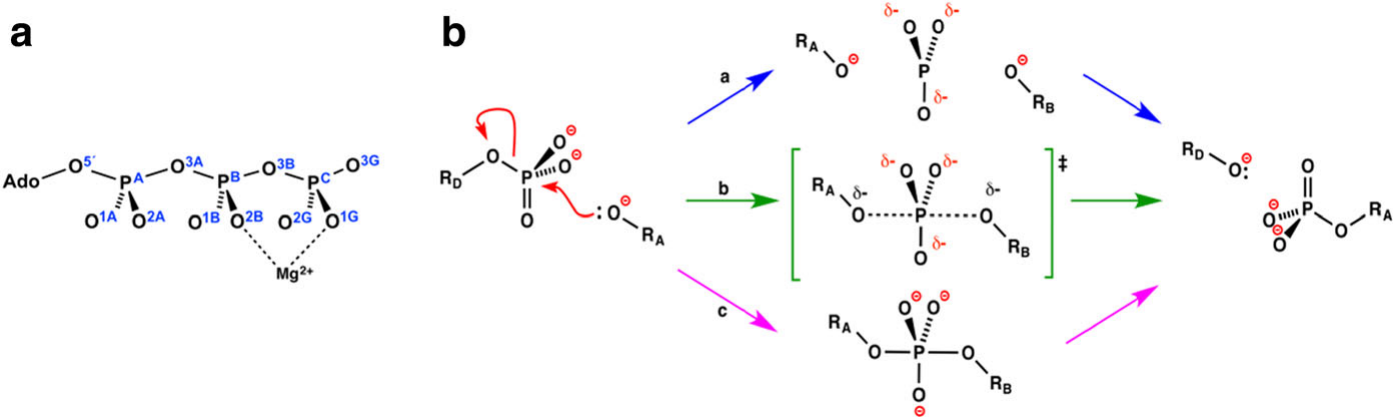
**a** The atomic identities in ATP employ the new IUPAC nomenclature [8]. **b** Three mechanisms for transfer of a phosphoryl group (PO_3_^–^, *top center*) between a donor (*left*) and an acceptor (*right*) species. Sequential process (**a**, *blue arrows*) involves formation of a trigonal planar metaphosphate anion as an intermediate. Concerted process (**b**, *green arrows*) shows a trigonal bipyramidal (tbp) transition state with phosphorus fully bonded to three equatorial oxygens and partially bonded to the axial donor (O_D_) and acceptor (O_A_) oxygens. An alternative, sequential process (**c**, *magenta arrows*), largely discarded, shows formation of a stable pentacoordinate phosphorane intermediate having full bonds to all five oxygens

Studies on phosphoryl transfer reactions were greatly advanced by the use of oxygen isotopes to show that they generally involved P–O cleavage, with transfer of the phosphoryl group (PO_3_^−^) between a donor oxygen (O_D_) and an acceptor oxygen (O_A_) or, less commonly, nitrogen or sulfur atoms [4]. Polyphosphates, such as ATP, react by attack of water (or an alcohol) on the terminal γ-phosphorus, breaking the P–O bond to the Ο^3Β^ atom (usually oxygen and infrequently nitrogen) (Scheme 1a). More advanced isotope work, deploying ^16^O, ^17^O, and ^18^O, established that the near-universal stereochemistry for such processes, for both chemical and enzymatic reactions, involves inversion of stereochemistry at the transferring phosphorus (Scheme 1) [5–7].

**Scheme 2:**
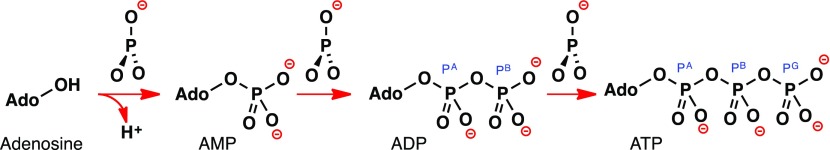
Successive capture of three phosphoryl groups, PO_3_^–^, converts an alcohol first into a phosphate monoester, then into an alkyl diphosphate, and finally into alkyl triphosphate, illustrated for adenosine 5′-triphosphate

An accurate description of the technical aspects of the varieties and uses of MF_*x*_ models calls for a brief explanation of the terminology of phosphoryl transfer. The phosphoryl group, PO_3_^–^ is identified throughout organic chemistry and biology as the anionic, trigonal planar assembly of a phosphorus and three oxygen atoms. It is usually drawn without P=O double bonds, is highly electrophilic, and has not been identified in any condensed phase (Scheme 2). It is helpful to perceive its combination with an alcohol, such as adenosyl-5′-OH, to generate a phosphate monoester, illustrated for adenosine 5′-phosphate, AMP. The addition of a second phosphoryl group to a terminal oxygen generates a pyrophosphate monoester, illustrated for adenosine 5′-diphosphate, ADP; and capture of a third phosphoryl group gives adenosine 5′-triphosphate, ATP (Scheme 2). Strings of phosphorus atoms in such chains have conventionally been labeled Pα, Pβ, Pγ, etc. but, with new IUPAC nomenclature, are now better identified as P^A^, P^B^, P^G^, P^D^, etc [8].

### Historic Development of Mechanisms

**Fig. 1:**
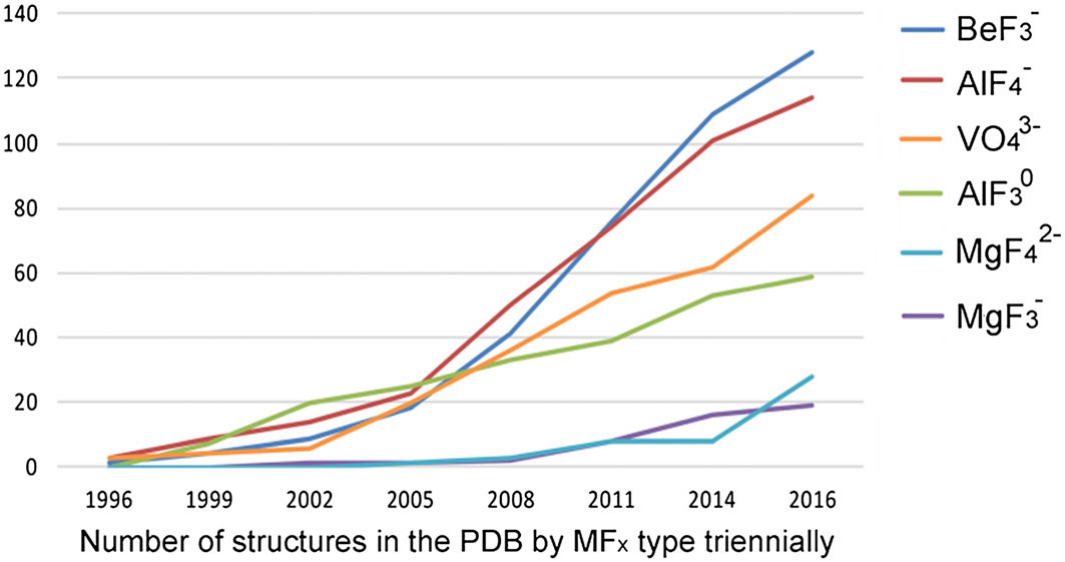
MF_*x*_ structures deposited in the Protein Data Bank since their inception in 1996. Vanadate (*orange*) is included for comparison

Todd [9] and Westheimer [2] thought that phosphoryl transfer reactions should be stepwise, involving a monomeric metaphosphate intermediate species (Scheme 1a). That concept, unproven after extended but fruitless effort, has now been discarded in favor of concerted phosphoryl transfer reactions for phosphate monoesters and anhydrides. These have “in-line” geometry for O_D_–P–O_A_ in the transition state (TS), with variable associative or dissociative character (Scheme 1b) [10–12]. Isotope labeling studies have contributed historically to studies on phosphoryl transfer in biological systems [13] and ^31^P NMR has been applied effectively for investigations on ATP [14] and phosphoarginine [15], but protein crystallography before the mid-1990s was restricted to binary complexes with stable substrate and bisubstrate analogs that gave limited information about reaction mechanisms [16, 17]. In 1994, that situation changed dramatically with the crystallization of ternary complexes of guanosine diphosphate (GDP) coordinated to tetrafluoroaluminate, AlF_4_^–^, and the small G protein, G_iα1_ (PDB: **1gfi**) [18] and with transducin α (PDB: **1tad**) [19]. Although these complexes had octahedral geometry for the aluminum tetrafluoride moiety, they were immediately described as transition state analogs (TSAs) for phosphoryl transfer. They were soon followed by further MF_*x*_ species, notably BeF_3_^–^, AlF_3_^0^, and MgF_3_^–^. The number of such complexes has grown steadily, now exceeding 350 (Fig. 1).

## Development of Metal Fluorides as Phosphate Analogs

### The ‘Burst Phase’ of Analog Discovery

**Fig. 2:**
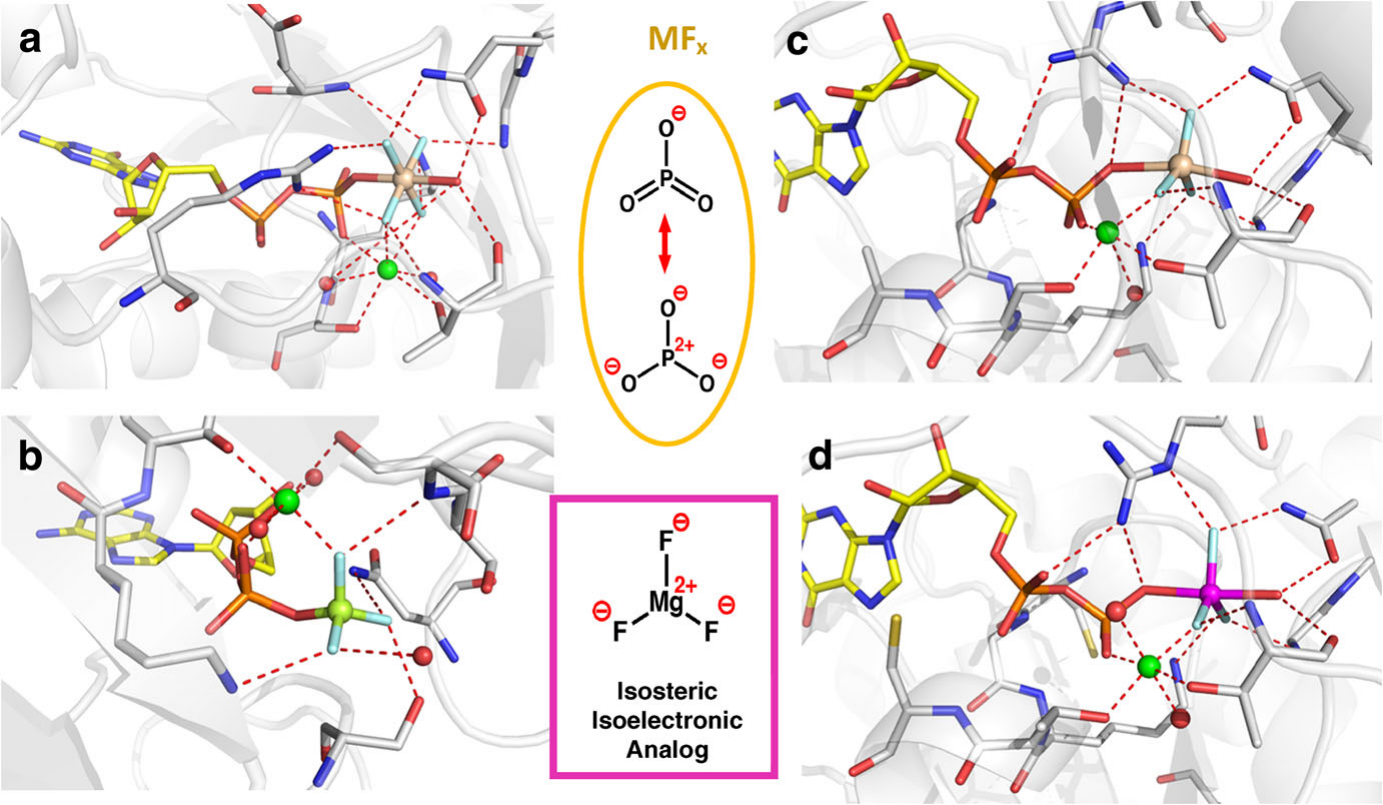
Landmark protein structures with a fourfold range of MF_*x*_ octahedral tetrafluoroaluminate, tbp aluminum trifluoride and trifluoromagnesate, and tetrahedral trifluoroberyllate complexes. **a** Transducin α with GDP coordinated to AlF_4_^–^ and catalytic magnesium (PDB: **1tad** at 1.7 Å). **b** Myosin with ADP coordinated to tetrahedral BeF_3_^–^ and Mg_cat_ (PDB: **1mmd** at 2.0 Å). **c** Ras·RasGAP with GDP coordinated to tbp AlF_3_^0^ and Mg_cat_ (PDB: **1wq1** at 2.5 Å). **d** RhoA·RhoGAP with GDP coordinated to tbp MgF_3_^–^ and Mg_cat_ (PDB: **1ow3** at 1.7 Å) (colors: aluminum, *grey*; beryllium, *lime*; surrogate magnesium, *magenta*; Mg_cat_, *green*; fluorine, *light blue*; nucleotides, *yellow*; key amino acids, *silver*; nitrogen, *blue*; oxygen, *red*)

Exciting developments in the field of signal switch mechanisms based on hydrolysis of GTP by small G proteins and on the molecular biology discoveries depending on ATP hydrolysis stimulated the development of three analog systems in the mid-1990s. Using tetrafluoroaluminate, work on G_iα1_, from the University of Texas, Dallas [18], narrowly edged out a closely related publication on Transducin α from Yale [19], while both focused on the role of the essential glutamine and arginine residues, of the catalytic magnesium, and on the positioning of water for attack on the terminal phosphate, P^G^ (Fig. 2a). Shortly after, a complex of ADP with AlF_4_^–^ was described to represent the TS for ATP hydrolysis in myosin, alongside the first structure of a trifluoroberyllate (BeF_3_^–^) complex with ADP, recognized as a ground state analog (GSA; Fig. 2b) [20].

After a short interval, a third class of MF_*x*_ analog was reported: an aluminum trifluoride complex of magnesium ADP for a dinucleotide kinase, described alongside the corresponding trifluoroberyllate tetrahedral complex. Its great advantage was tbp geometry for the TSA complex that, for the first time, accurately mimicked the TS geometry of the γ-phosphate of ATP undergoing transfer [21]. This was quickly followed by a GDP·AlF_3_^0^ complex for the small G protein Ras·RasGAP (Fig. 2c) [22] and then by an ADP·AlF_3_^0^·GDP complex for a quaternary complex of a nucleoside diphosphate kinase from the slime mold*, Dictyostelium discoideum* [23]. These, and subsequent examples of tbp complexes, recognized that AlF_3_^0^ was a neutral MF_*x*_ species and therefore a Coulombic mismatch for an *anionic* phosphoryl group. It was 5 years before that feature was rectified with the first identification of trifluoromagnesate (MgF_3_^–^) bound to GDP in a complex with the small G protein, RhoA. A key component of that work was the rigorous use of proton-induced X-ray emission spectroscopy (PIXE) to identify magnesium as the atom at the core of the tbp complex (Fig. 2d) [24].

By this time, there were some 50 structures deposited in the PDB for MF_*x*_ complexes, usually with anionic oxygen as one axial ligand. Their importance has stimulated a rapid, ongoing growth in their use (Fig. 1). We shall now examine the relative qualities of these four classes and their offshoots on a systematic basis, organized by geometric considerations.

## MF_*x*_ Ground State Analogs

### BeF_3_^–^ as a Ground State Phosphate Mimic

In aqueous solution, beryllium (II) forms stable fluorides as a mixture of tetrahedral species including BeF_2_·2H_2_O, BeF_3_^–^·H_2_O, and BeF_4_^=^ [25]. ^19^F NMR studies on fluoroberyllate complexes with ADP identified mixed fluoroberyllate·ADP species for myosin (Fig. 2b). Nearly 130 trifluoroberyllate complexes have now been described, with three structures solved by NMR and 119 X-ray structures having resolutions of ≥1.2 Å, generally having tetrahedral trifluoroberyllate bonded to an anionic oxygen. These comprise two sub-groups: over 70 have Be coordinated to an aspartate carboxylate while some 50 have Be coordinated to a terminal phosphate oxygen of a nucleotide. Just two have Be coordinated to a histidine ring nitrogen, while one has BeF_2_ bridging two phosphates.

#### Aspartyl Trifluoroberyllates

**Fig. 3:**
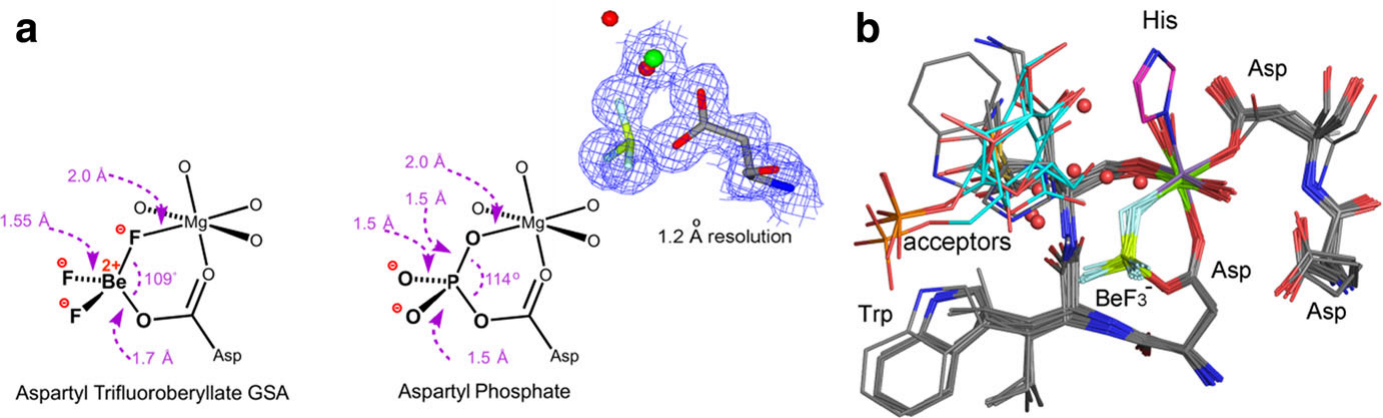
**a** Typical aspartyl trifluoroberyllate structure with catalytic magnesium coordination (*left*). Aspartyl phosphate complex with catalytic magnesium from phosphoserine phosphatase (PSP) (PDB: **1j97**) for comparison of geometry (*center*). Electron density map for the 1.2-Å resolution structure for β-phosphoglucomutase (PDB: **2wf8**) (*center*). **b** Twenty aligned aspartyl–trifluoroberyllate structures with BeF_3_^–^ locked in a six-membered ring. Catalytic Mg^2+^ and an Asp carboxylate fuse a 13-atom ring to the fluoroberyllate ring (*rear*). Octahedral coordination to Mg is completed by an additional aspartate (*right*), by 1–2 waters, and only twice by a histidine (*top*, *magenta*) (atom colors: fluorine, *light blue*; beryllium, *lime*; nitrogen, *blue*; oxygen, *red*; carbon, *grey*; 3-phosphoglycerate, *cyan*) (electron densities presented in CCP4MG from mtz data in EDS and contoured at 1σ) (**a** adapted by the authors from [27])

Aspartyl phosphates are intermediates in many enzyme reactions, with a half-life for spontaneous hydrolysis from 23 s to a few hours [26]. Aspartyl trifluoroberyllates are stable and available for analysis by ^19^F NMR and protein crystallography. They are tetrahedral, ground state mimics of an aspartyl phosphate. The 70 structures have a common core, with bidentate coordination to a divalent metal ion, generally Mg^2+^ and rarely Mn^2+^, from fluorine F_1_ and the second carboxylate oxygen to give a near-planar six-membered ring (Fig. 3a; all MF_*x*_ structural data are tabulated in a recent review [27]). Because of its low electron density, the beryllium atom is difficult to locate by X-ray diffraction, resulting in uncertainty in its exact position, leading to considerable variation in attributed geometry (Fig. 3b): the 27 best resolved structures have a Be–O distance 1.72 Å with Be–F 1.53 Å.

#### BeF_3_^–^ Nucleotide Structures

**Fig. 4:**
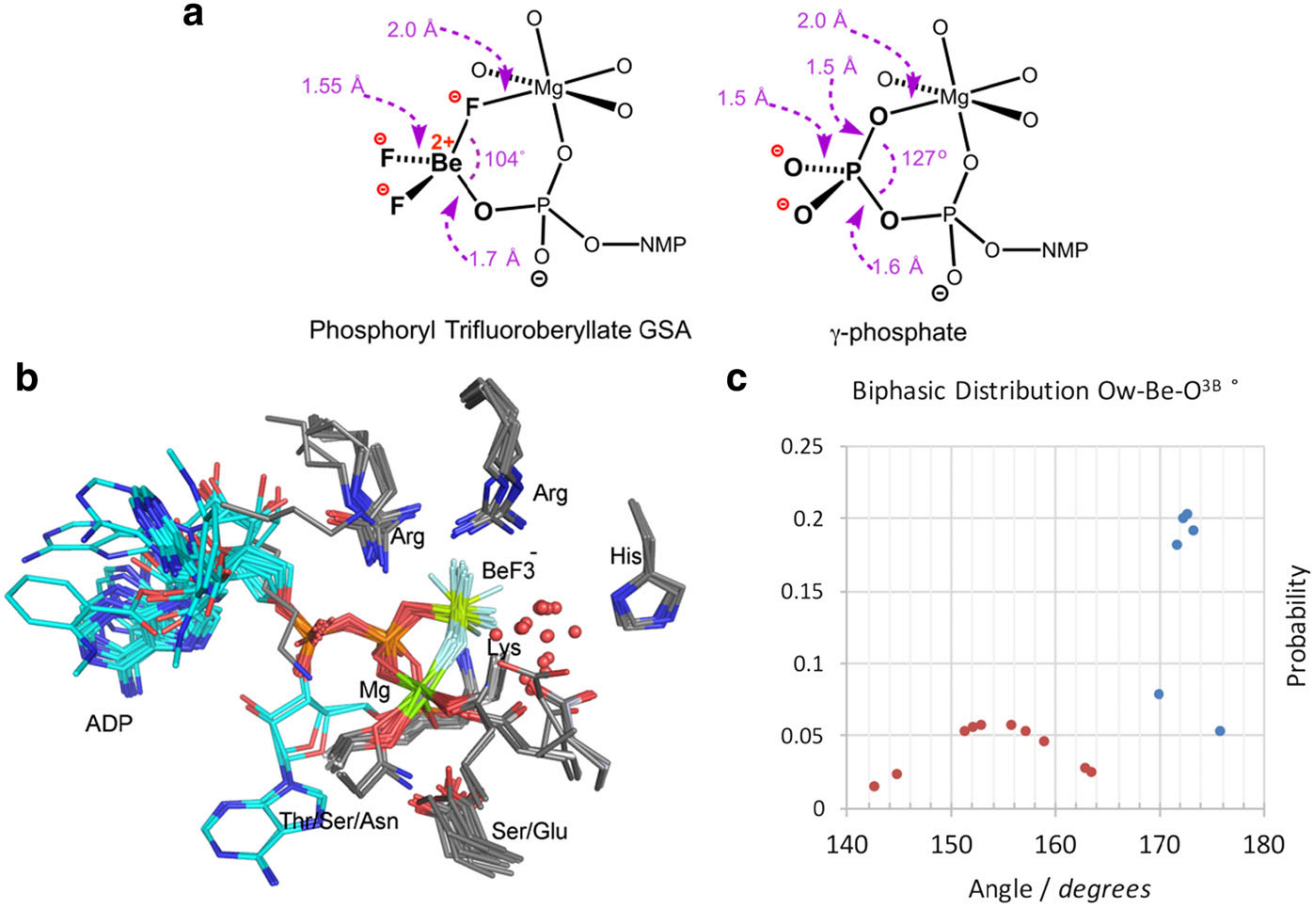
**a** Typical nucleoside diphosphate trifluoroberyllate structure (*left*) with catalytic magnesium coordination for comparison of geometry with the nucleoside triphosphate (*right*). **b** The BeF_3_^–^ moiety in 20 aligned ADP·trifluoroberyllate structures is in a six-membered ring (*center*) with Mg^2+^ coordinating F_1_ and O^3B^. γ-Phosphate coordination to an Arg and a Lys is also common. **c** Biphasic normal distribution of the location of the nucleophilic water, Ow, relative to the bond from ADP–O^3Β^ to beryllium in 16 ADP·BeF_3_^–^ ground state complexes. Major group Ow–Be–O^3B^ angle ≥165˚ (*orange*); minor group Ow–Be–O^3B^ angle 176˚ ≥170˚ (*blue*)

There are 42 X-ray structures of BeF_3_^–^ complexes with ADP, and six with GDP. They are isosteric mimics of ATP and GTP (Fig. 4a) in kinases, F1 ATPase, hydrolases, mutases, helicases, and small G proteins. Twenty structures align very well (Fig. 4b) with Be bonded to a β-phosphate oxygen, while a catalytic Mg^2+^ is coordinated to F_1_ and to another β-phosphate oxygen.

#### Histidine Trifluoroberyllates

**Fig. 5:**
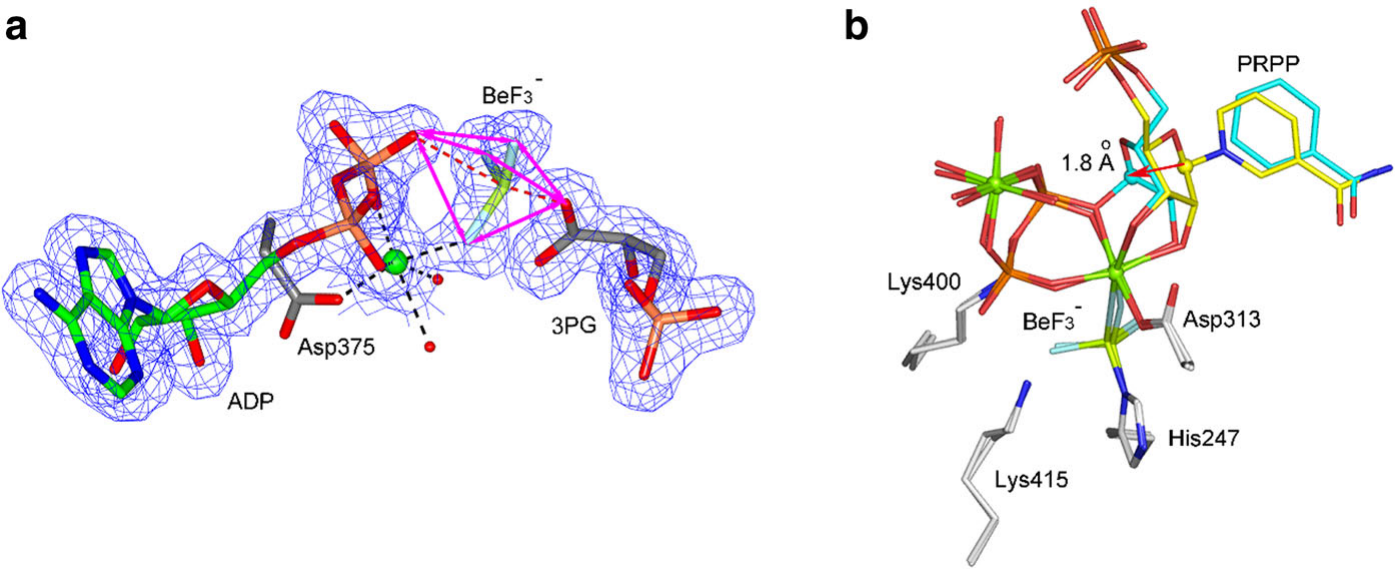
**a** Structure of BeF_3_^–^ complex for βPGK (PDB: **4axx**). Beryllium (*lime green*) is “in-line” between a Ο^3Β^ of ADP and 3PG. **b** Nicotinamide phosphoribosyl transferase (PDB: **3dhf**) catalyses displacement of pyrophosphate from C1 of ribose 5-phosphate. Structures of two overlaid complexes show BeF_3_^−^ bound to Nη of His247 and one fluorine coordinating Mg^2+^ (*green sphere*). PRPP reactant C1’ (*cyan sphere*) moves 1.8 Å to bond to nicotinamide N1 (atom colors: fluorine, *light blue*; beryllium, *lime*; nitrogen, *blue*; oxygen, *red*; protein residues are in *grey*; nucleotides in *cyan*) (**a** is reproduced from [27])

Various approaches to analogs of η-phosphohistidine have been explored. Structural work on nicotinamide phosphoribosyltransferase (NAMPT) has mimicked phosphorylation of an active-site histidine using trifluoroberyllate. Crystal structures of NAMPT for reactant and product complexes (PDB: **3dhf**; Fig. 5b) have a covalent His247·BeF_3_^−^, and in contrast to all other trifluoroberyllate structures, magnesium is coordinated to one fluorine without any direct linkage to His247 [28].

#### Structural Conclusions

The significant ability of beryllium (II) fluorides to complete tetrahedral coordination by binding to an anionic oxygen has made them good isosteric and electrostatic GSAs of phosphate for a wide range of uses [29]. Bond lengths for Be–F and Be–O are close to those for P–O (1.6 ± 0.5 Å) and the strong ionic character of the Be–F bond means that its fluorines readily accept H-bonds from a range of donors and/or coordinate to Group 2 metal ions [30]. Thus, fluoroberyllates have been used beneficially to study changes in major conformations of proteins by crystallography, NMR, and EM, while studies on ADP**·**BeF_3_^–^ have supported investigations on ATPases that drive various mechanical processes at a molecular level, particularly for myosin [31–36]. They have proved especially valuable for the identification of near attack conformations (NACs) in enzyme mechanisms, notably for β-phosphoglucomutase (βPGM) [37].

## MF_*x*_ in Transition State Analog Complexes

### Tetrafluoroaluminate TS Complexes—AlF_4_^–^

**Fig. 6:**
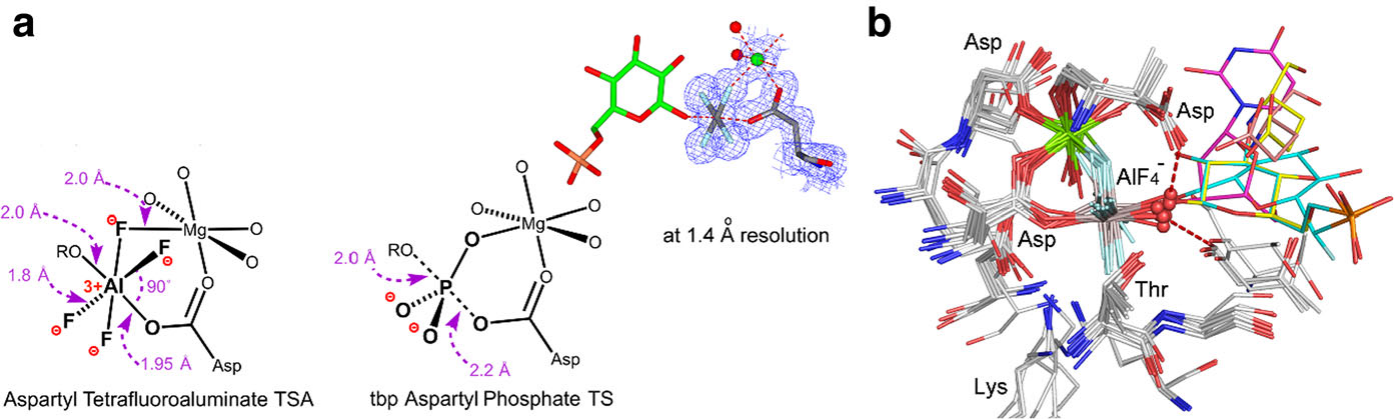
**a** Typical aspartyl tetrafluoroaluminate structure with catalytic magnesium coordination (*left*). Aspartyl phosphate complex with catalytic magnesium from phosphoserine phosphatase (PDB: **1j97**) for comparison of geometry (*center*). Electron density map for the 1.2-Å resolution structure for β-phosphoglucomutase (PDB: **2wf8**) (*right*). **b** Structures of 13 aspartyl tetrafluoroaluminates aligned on Cα. Octahedral aluminum is coordinated to Asp-O^4^, in a six-membered ring with Mg_cat_ and “in-line” with a nucleophilic water oxygen (*red sphere*) or the OH group of a nucleoside or hexose reactant (*rainbow colors*) (atom colors: fluorine, *light blue*; aluminum, *grey*; nitrogen, *blue*; oxygen, *red*; magnesium, *green sphere*) (Figure reproduced from [27])

Aluminum (III) forms stable fluorides in water, the mixture of octahedral species including AlF_2_^+^·4H_2_O, AlF_3_·3H_2_O, AlF_4_^–^·2H_2_O, and AlF_5_^=^·H_2_O, depending on the concentration of fluoride [38, 39]. Crystal structures for octahedral GDP·AlF_4_^–^ protein complexes [18–20] were prompted by the discovery that aluminum plus fluoride stimulates the activity of small G proteins in the presence of GDP [40], while ^19^F NMR analysis of a GDP·AlF_*x*_ complex for G_1_α [41] confirmed that they could mimic bound GTP [42]. All 114 crystallographic AlF_4_^–^ complex structures in the PDB (PDB ligand: **ALF**) are octahedral and have aluminum sandwiched between donor and acceptor atoms, predominantly oxygens. Unlike beryllium, the aluminum is well defined in the electron density map (Fig. 6a) and can accept a neutral oxygen as one axial ligand. However, aluminum forms insoluble Al(OH)_3_ above pH 7.5 [38, 39], which restricts the stability of aluminum fluoride complexes to pH <8.

#### Aspartyl Tetrafluoroaluminates

Fourteen PDB structures have tetrafluoroaluminate bonded to an aspartate with an essential Mg^2+^ in a six-membered ring. They align well on the best resolved complex, β-phosphoglucomutase (βPGM, PDB: **2wf7**; 1.05-Å resolution (Fig. 6b), with four equatorial oxygen ligands coordinating the catalytic Mg^2+^. The structures fit into two subsets: six members of the first group have a second aspartate sub-adjacent to the first (Asp8 and Asp10 in βPGM). The O_A_–Al–O_D_ bonds are “in-line” (167.5˚ ± 7.0˚) with the aluminum midway between the two oxygens (separation 3.9 ± 0.1 Å) and have a catalytic aspartate that accepts a short H-bond from the apical water/hydroxyl group (2.59 ± 0.05 Å) to align this oxygen for nucleophilic attack on phosphorus [43].

The second subset has ATPases involved in pumping calcium, copper, and zinc ions. They use an aspartyl phosphate intermediate, whose TS for hydrolysis is mimicked by an octahedral AlF_4_^–^. An axial water oxygen forms short H-bonds to an invariant glutamate (2.5 ± 0.1 Å) and to a threonine carbonyl (2.57 ± 0.05 Å), which clearly orientate and polarize the water for “in-line” attack on the aspartyl phosphate [44].

#### Nucleotide Guanosine Diphosphate (GDP) Tetrafluoroaluminates

**Fig. 7:**
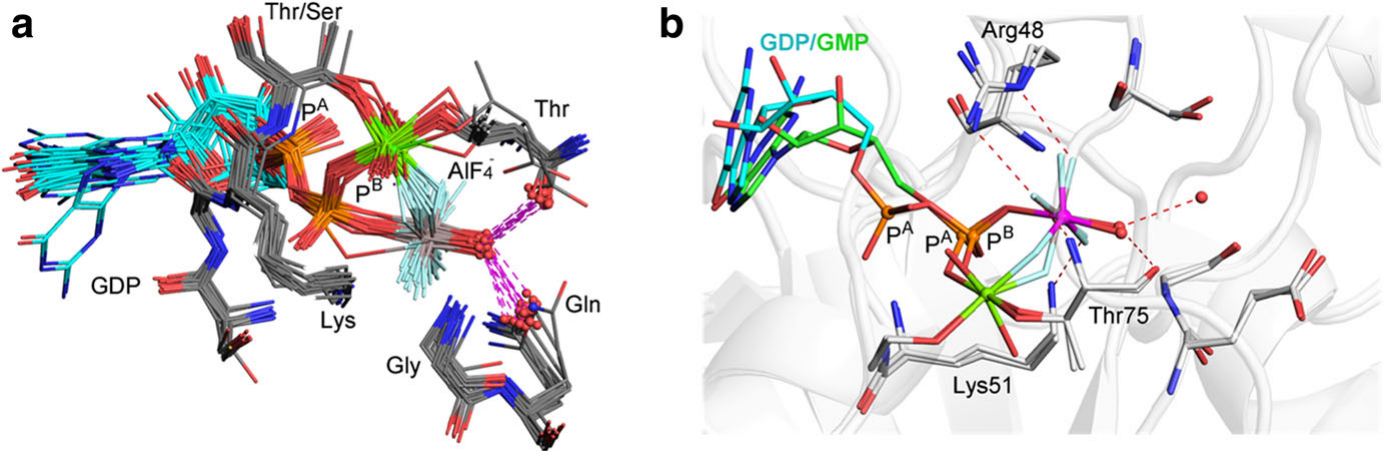
**a** Twenty GDP·AlF_4_^–^ structures aligned on α-carbon atoms of the invariant hexapeptide (in PDB: **2gj8**). AlF_4_^–^ is locked in a six–membered ring (*center*) with Mg_cat_ (*green spheres*) coordinating F_1_ and a P^B^ oxygen. Octahedral coordination to Mg^2+^ is provided by a second P^B^ oxygen, two waters, a Thr hydroxyl (*right*), and a Ser/Thr hydroxyl (*top*). P^B,G^ oxygens H-bond to a Lys (*center*). **b** Structures of hGBP1 with a GMP·AlF_3_^0^ complex (*cyan*) aligned with a GDP·AlF_4_^–^ complex (*green*) showing occupancy of the catalytic site by the AlF_3_^0^ mimic of P^B^ (*magenta sphere*) and by the AlF_4_^–^ mimic of P^G^ (*grey sphere*) (atom colors: GDP, *cyan*; GMP, *green*; magnesium, *green*; fluorine, *light blue*; amino acids, *silver*; nitrogen, *blue*; oxygen, *red*)

GDP forms 50 AlF_4_^–^ complexes that constitute isoelectronic but non-isosteric mimics of GTP in a broad range of proteins. The best resolved 21 align remarkably well (Fig. 7a), with aluminum bonded to O^3B^ on GDP and the Mg_cat_ coordinated to F_1_ and O^1B^ in a six-membered ring. The guanosine base and ribose usually occupy a common conformation (Fig. 7a). The geometry of the AlF_4_^–^ moiety is regularly octahedral, with “in-line” O_A_–Al–O_D_ angle 172.8˚ ± 7.1˚. All structures have an axial oxygen ligand (Fig. 7a, red spheres) coordinated to aluminum that is trigonal planar with respect to two H-bond acceptors: the backbone carbonyls of a threonine and a glutamine side-chain (occasionally a water) (Fig. 7a, lower right, red spheres).

#### Nucleotide Adenosine Diphosphate (ADP) Tetrafluoroaluminates

**Fig. 8:**
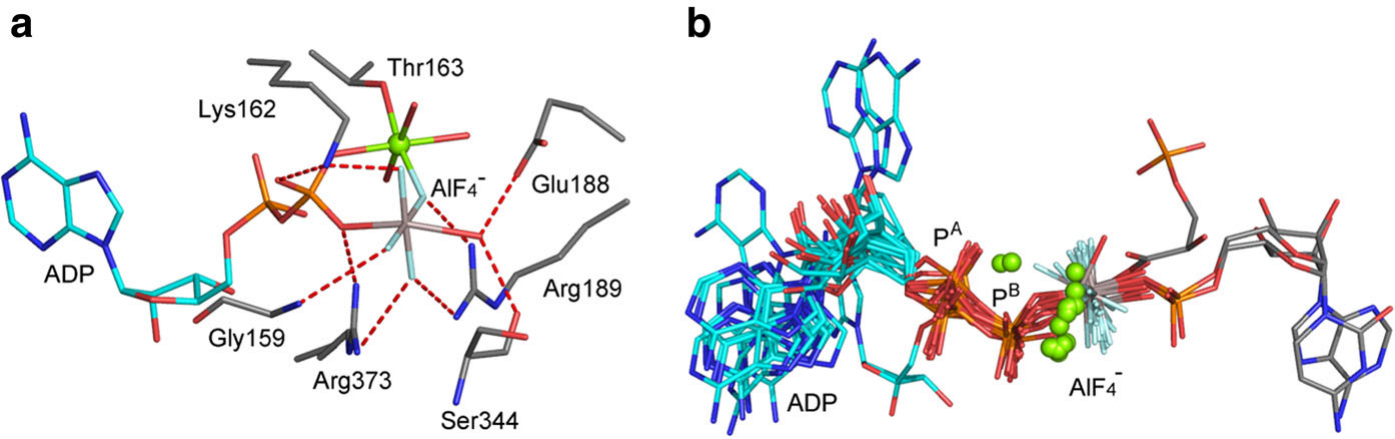
**a** F1 ATPase TSA complex **(PDB: 1h8e)** with ADP·AlF_4_^–^ showing local charge balance for five **+**ve and five −ve charges. **b** 16 ADP·AlF_4_^–^ complexes aligned for C5’, P^A^, P^B^, and Al show great variety in ATP analog conformations (atom colors: adenosines, *cyan*; magnesium, *green spheres*; fluorine, *light blue*; aluminum, *gold*; amino acids and second substrates, *grey*; nitrogen, *blue*; oxygen, *red*)

Forty-nine octahedral structures have AlF_4_^–^ bonded to a terminal oxygen of ADP (O^3B^) to mimic ATP in the TS. They are found in kinases, hydrolases, isomerases, ATPases, myosins, helicases, transporter pumps, and nitrogenase. The 31 that are best resolved have an axial O_A_–Al–O_D_ distance of 4.05 ± 0.03 Å with an “in-line” angle of 170˚ ± 8˚ and most have water as the second oxygen ligand with a catalytic Mg^2+^ coordinating one of the fluorines. This is illustrated for F1 ATPase (PDB: **1h8e**) (Fig. 8a). In contrast to the uniform conformation for complexes with GDP (Fig. 7), complexes with ADP show a great variety of conformations, as illustrated for 16 well-resolved structures (Fig. 8b).

### Octahedral Aluminum Trifluoride Phosphate TS Mimics

**Fig. 9:**
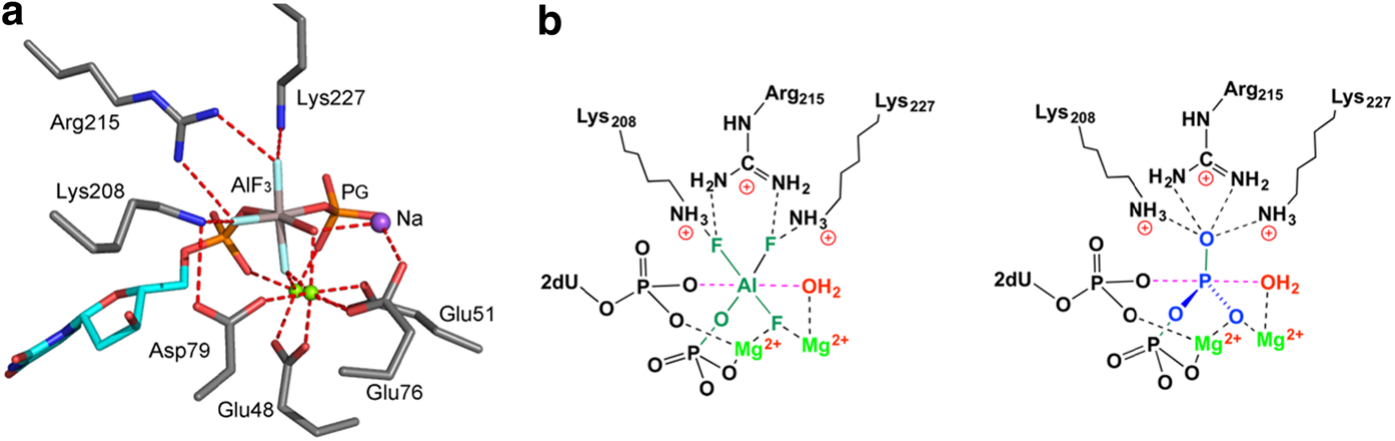
**a** Aluminum trifluoride structure for dUTPase (PDB: **4di8**). UMP (*cyan*) coordinates aluminum (*grey*) with in-line water (*red*) and with PO_4_^=^ adjacent to the leaving O^3A^. Two magnesiums (*green spheres*) are located by coordination to the reactants and to four carboxylate residues (amino acids in *grey*). **b** Cartoon showing octahedral aluminum trifluoride sharing the tbp coordination of the true TS for a phosphoryl group (colors: nucleoside, *cyan*; magnesium, *green sphere*; aluminum, *grey*; sodium, *purple*; amino acids, *silver*; nitrogen, *blue*; oxygen, *red*) (Figure adapted from [27])

An aluminum trifluoride moiety accepts three oxygens to give an octahedral, six-coordination TSA complex in three examples. In the small G protein Rab5a, the mutation A30P enables addition of the side chain hydroxyl of Ser29 to aluminum trifluoride (PDB: **1n6k**). In the case of hPGK, the K219A mutant has a water as the fourth ligand coordinated to the aluminum [45]. Thirdly, for a bacterial dUTPase, aluminum trifluoride takes the place of the P^B^ in dUTP with coordination to two oxygens from the β-phosphoryl group and to the water nucleophile to complete the octahedral array (Fig. 9a, b). This significant structure provides a unique example where nucleophilic attack is directed at a non-terminal nucleotide phosphorus [46].

## MF_3_ Improved Geometry Transition State Mimics

### MgF_3_^–^, Trifluoromagnesate

**Fig. 10:**
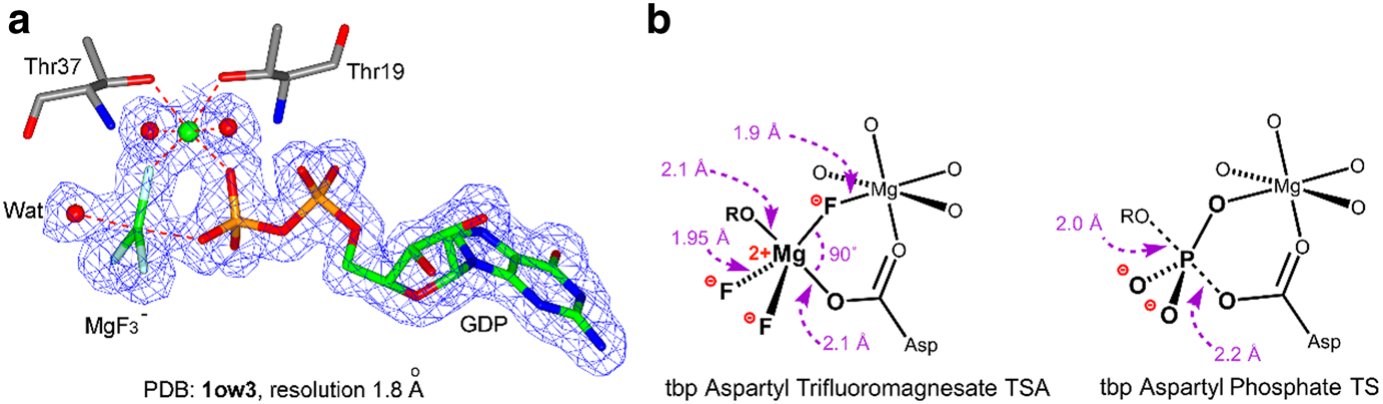
**a** MgF_3_^–^ complex with GDP for RhoA/GAP (PDB: **1ow3**) showing electron density. **b** Typical MgF_3_^–^ complex with aspartate residues in a six-membered ring with the catalytic Mg^2+^ (*left*) compared to an aspartyl phosphate (*right*) (Figure adapted from [27])

Magnesium does not form mixtures of stable fluorides in water at sub-molar concentration: only one resonance for magnesium fluoride is seen in ^19^F NMR solution spectra, that of MgF^+^. While MgF_2_ is moderately soluble in water (2 mM), it has an estimated dissociation constant of 10^−5^ M [47]. Trifluoromagnesate protein complexes were first anticipated based on magnesium-dependent fluoride inhibition studies, and they led directly to the identification of MgF_3_^–^ in a tbp crystalline TSA complex for the small G protein RhoA·RhoGAP (Fig. 10a) [24, 48]. The PDB now has 16 entries for trifluoromagnesate (PDB ligand: **MGF**) while a further three entries assigned as tbp AlF_3_^0^ have been shown by ^19^F NMR to be MgF_3_^–^ complexes [49–51]. Standard coordination chemistry identifies magnesium as being regularly octahedral, forming complexes with six (oxygen) ligands. By contrast, trifluoromagnesate in protein complexes is unexpectedly five-coordinate. This makes it ideal for mimicking tbp phosphoryl transfer and, moreover, *MgF*_*3*_^–^ *is isoelectronic with PO*_*3*_^–^. Examples of its use include complexes of small and large molecule kinases, mutases, phosphatases, and hydrolases, which invariably involve fluorine coordination to a catalytic Mg^2+^ (two magnesiums in the case of some protein kinases). These are usually octahedral and built into a cyclic six-membered ring structure, as shown for aspartyl phosphate mimics (Fig. 10b). They have an axial O_**A**_–Mg–O_**D**_ distance of 4.19 ± 0.08 Å with an in-line angle 171.4˚ ± 3.9˚.

### AlF_3_^0^, Aluminum Trifluoride

**Fig. 11:**
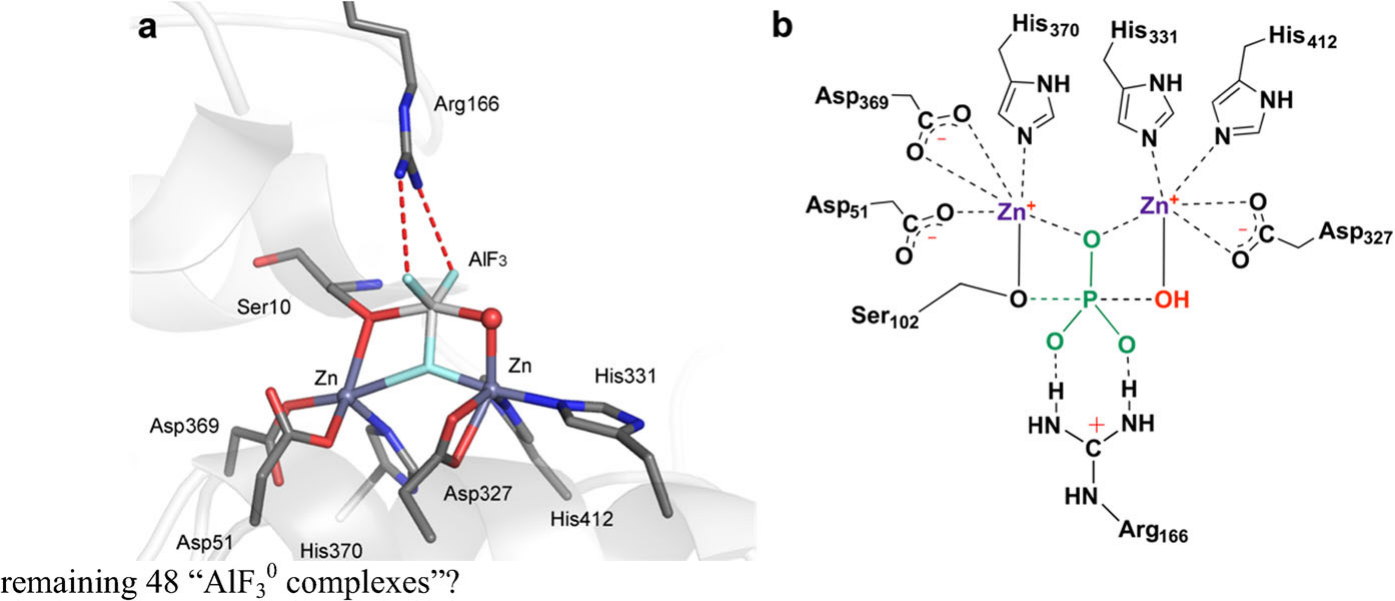
**a** Structure of the catalytic center for alkaline phosphatase complexed to AlF_3_ (PDB: **1kh5**). **b** Cartoon of the coordination organization in the active site with transferring phosphoryl group (*blue*) and nucleophilic water (*red*) (Figure adapted from [27])

There are now 56 examples of structures purported to have an AlF_3_^0^ core. Three of them are octahedral, while ^19^F NMR has established that another three are MgF_3_^–^. For the remaining majority, only structures of two alkaline phosphatase complexes (AP) can be confidently identified as having a tbp AlF_3_^0^ core (Fig. 11). One is in mutant AP_P300A_ (PDB: **1kh5**), where two catalytic Zn^2+^ ions coordinate one fluorine while Ser102 and a zinc-coordinated water provide the axial ligands for the tbp aluminum (Fig. 11b). What about the remaining 48 “AlF_3_^0^ complexes”?

**Fig. 12:**
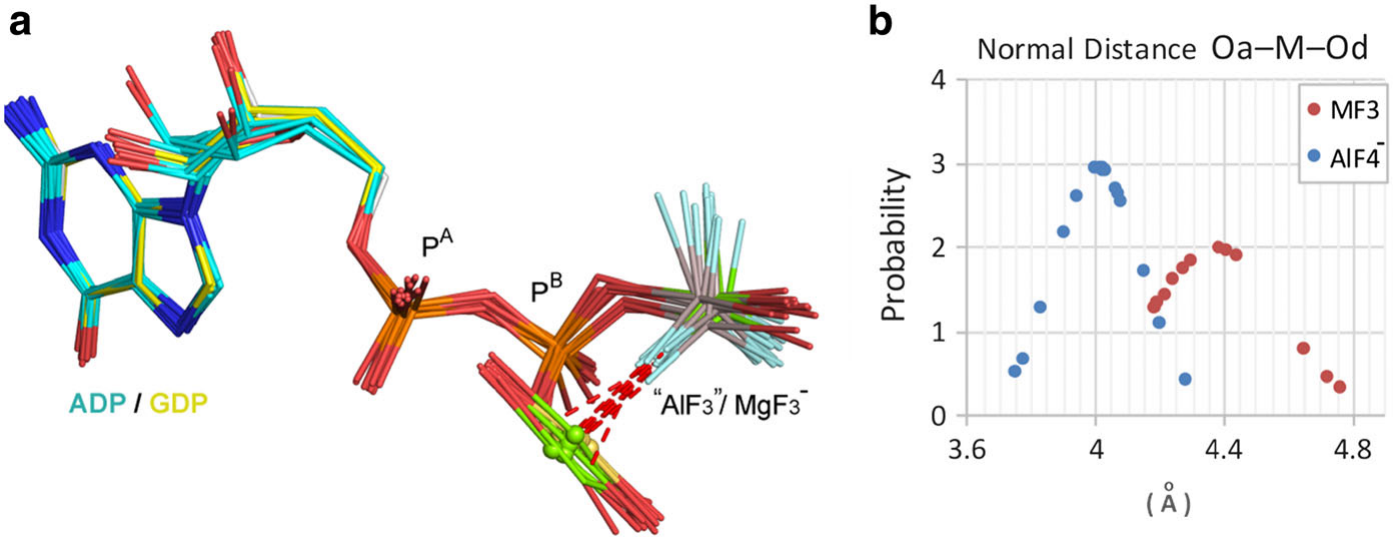
**a** Overlay of five GDP·MgF_3_^–^ (*yellow*) and eight GDP·AlF_3_^0^ (*cyan*) complexes to show the geometric uniformity of the two sets of TSA structures. **b** Normal distribution plots for the O_A_–M–O_D_ distance for this set of 13 structures (*red*) and the corresponding O_A_–Al–O_D_ distance for 18 GDP·AlF_4_^–^ TSA complexes (*blue*)

The pH dependency of the transition between octahedral and tbp structures of AlF_*x*_ complexes in protein crystal structures was proposed to involve a switch from AlF_4_^–^ to AlF_3_^0^ at elevated pH [52]. However, studies on the pH dependence of the solubility of aluminum ion [38, 39] provided an alternative interpretation. Al(OH)_3_ precipitates at pH ≥8, which results in aluminum being superseded by magnesium in protein MF_*x*_ complexes at high pH, with a consequent change in geometry from octahedral to tbp. That conclusion has now been validated by pH-dependent ^19^F NMR analyses for several enzymes [50, 53]. In some marginal cases, e.g., protein kinase A (cAPK) and PSP, there is mixed occupancy of the active site by tbp and octahedral complexes in the crystal [43, 50, 51]. In geometric terms, “AlF_3_^0^” tbp complexes closely map on those of trifluoromagnesates: axial O_A_–M–O_D_ bonds 4.29 ± 0.39 Å (Fig. 12b), and M–F bonds 1.75 ± 0.12 Å. It seems likely that some, or many, of these “AlF_3_^0^” complexes are trifluoromagnesates: a conclusion supported by geometric analysis for both families of complex.

### A Combined MgF_3_^−^- and AlF_3_^0^ Structural Analysis

A statistical analysis of the structures of AlF_3_^0^ and MgF_3_^−^ complexes contributes to the resolution of this compositional uncertainty. The near-invariant geometry of octahedral AlF_4_^−^ complexes for GDP makes them a useful set for comparison with the corresponding set of tbp MF_3_ complexes. Thus, eight GDP “AlF_3_^0^” structures for small G proteins align very well with those for five MgF_3_^−^ complexes (Fig. 12a). The axial separation for the donor and acceptor oxygens in these combined 13 GDP·MF_3_ TSAs is 4.38 ± 0.20 Å, significantly distinct from the corresponding average for 19 GDP·AlF_4_^–^ complexes, 4.02 ± 0.14 Å, and clearly supported by normal distribution analysis (Fig. 12b). The conclusion is: For “AlF_3_^0^” read MgF_3_^–^!

Taking “AlF_3_^0^” together with trifluoromagnesates, a common general pattern of axial ligands emerges. The MF_3_ species requires at least one anionic oxygen. β-Oxygens from ADP (33 structures) and GDP (24 structures) provide the overwhelming majority of examples while aspartate (11 structures) is also significant. Water (27 structures) is the dominant neutral axial ligand while serine and threonine hydroxyls appear less frequently. Significantly, there is no example of both axial ligand positions being occupied by two neutral ROH groups.

### MgF_4_^=^, Tetrafluoromagnesate

**Fig. 13:**
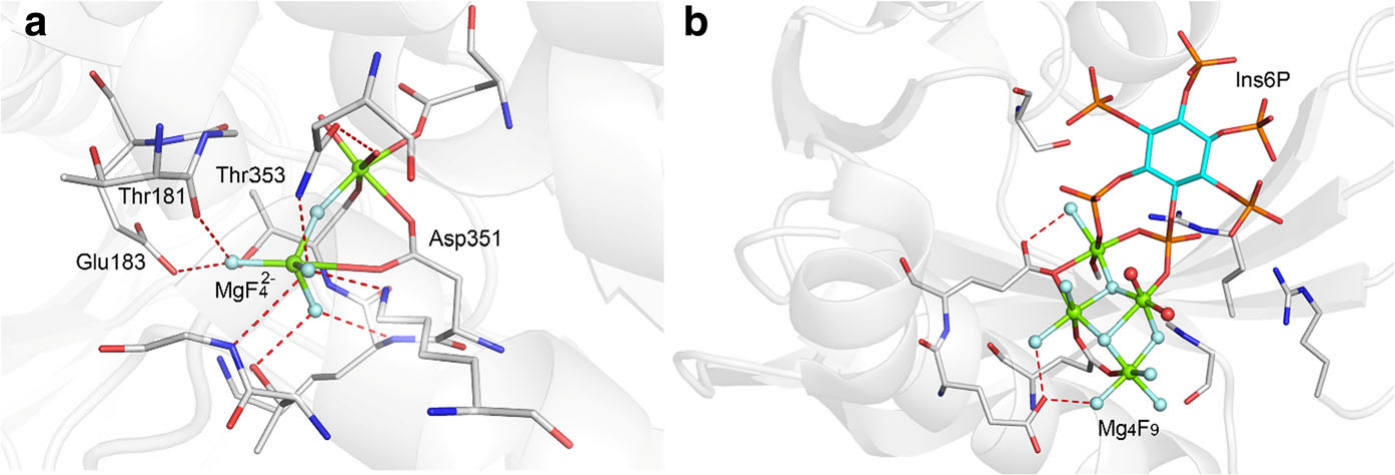
**a** Structure of Ca^2+^ pump ATPase with MgF_4_^=^ (PDB: **1wpg**). Coordination for MgF_4_^=^ is typical of an aspartyl trifluoromagnesate complex (colors: fluorine, *light blue*; magnesium, *green*; nitrogen, *blue*, oxygen, *red*; carbons, *silver*). **b** Structure of hPPIP5K2 (PDB: **2q9p**) to show the “Mg_4_F_9_” cluster adjacent to phosphates 4 and 5 of Ins6P

There are several structures for the Ca^2+^ pump ATPase that have been assigned as tetrahedral MgF_4_^=^ moieties without objective experimental validation. Magnesium is only exceptionally four-coordinate and then it usually has sterically bulky ether oxygens as ligands [54]. The tetrahedral “MgF_4_^=^” moiety in all PDB examples is remote from ADP, is coordinated to a second magnesium, and has one or more of its four “fluorine” atoms in close contact with a backbone carbonyl oxygen, as shown for PDB: **1wpg** (Fig. 13a) [55]. Such “MgF_4_^=^” behavior closely resembles the six-membered ring tbp structures common for MgF_3_^–^ complexes of aspartate (Fig. 10). Crystallographic re-refinement, with MgF_3_^–^ replacing MgF_4_^=^ for **1wpg**, can produce an equally valid structure. Thus, unless established by further measurements, a more consistent chemical interpretation for all such “MgF_4_^=^” situations is that they are trifluoromagnesates that mimic the TS for hydrolysis of an aspartyl phosphate. Subsequent work has described a similar tetrahedral moiety for the Na^+^/K^+^ pump ATPase (PDB: **2zxe**) [56].

Finally, the most remarkable MF_*x*_ structure is that of a human diphosphoinositol phosphatase, co-crystallized with *myo*-inositol *hexakis*-phosphate and then soaked with sodium fluoride (PDB: **2q9p**) [57]. This complex has four octahedral magnesiums with nine ligands assigned as fluorines in a complex that embraces MgF_2_, MgF_3_, MgF_4_, and MgF_5_ species in a single block. It also offers the first example of octahedral MgF_*x*_ (Fig. 13b). Its core appears related to the Rutile structure of MgF_2_, which is characterized by octahedral magnesium and trigonal planar fluorine [58].

## ^19^F NMR Studies of MF_*x*_ Complexes

**Fig. 14:**
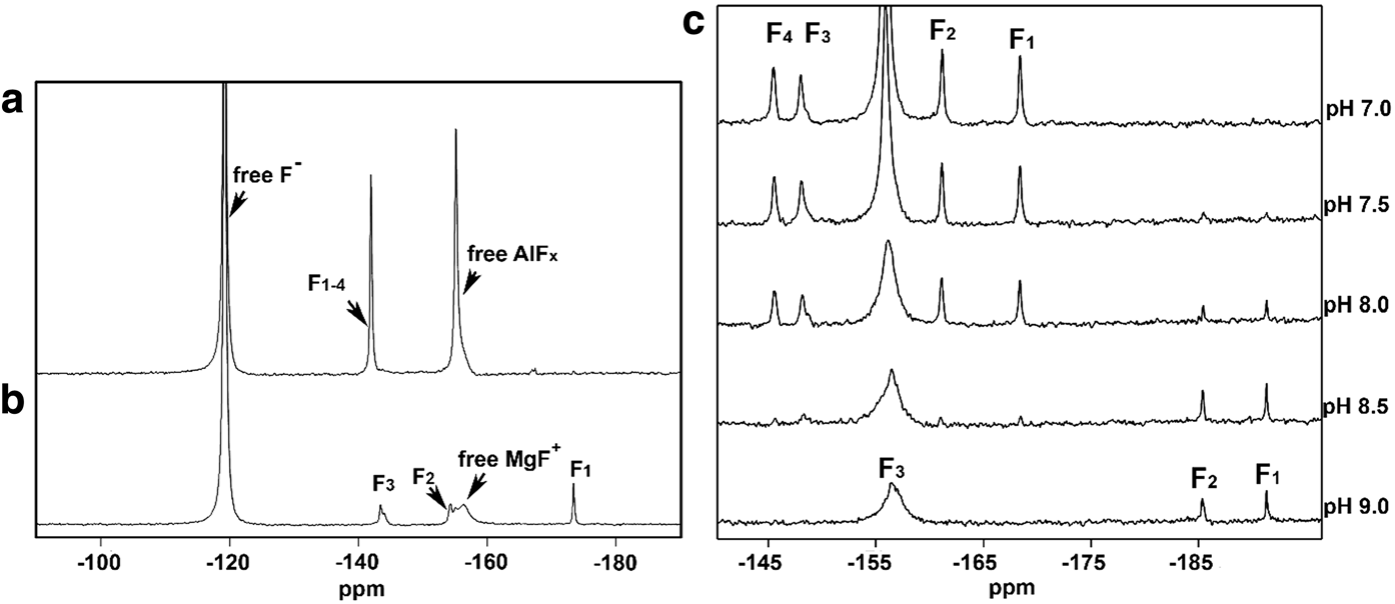
**a** 1D ^19^F NMR spectra of: **a** RhoA/GAP·GDP·AlF_4_^–^ TSA complex with four fluorines rotationally averaged [44, 48]. **b** RhoA/GAP·GDP·MgF_3_^–^ TSA complex with three fluorines resolved [44]. **c** Conversion of the cAPK·ADP·AlF_4_^–^ TSA complex to the cAPK·ADP·MgF_3_^–^ TSA complex in a pH titration from pH 7.0 to pH 9.0 [50]. The ^19^F resonance at −119 ppm in each spectrum is from free F^–^ ion, while the broad peaks around −156 ppm are from unbound MgF^+^ and AlF_*x*_ species, which may often overlap with protein bound fluorine resonances. In those cases, presaturation of the free fluoride resonance at −120 ppm can be applied to eliminate the unbound metal fluoride signals

The very high gyromagnetic ratio (25.18 × 10^7^ T^−1^ s^−1^) of ^19^F gives it very high sensitivity in NMR, which facilitates detection of fluorine-containing species at low concentration in large molecular weight complexes, as illustrated for AlF_4_^–^ and MgF_3_^–^ complexes with RhoA**·**GAP**·**GDP (Fig. 14a, b) [44, 50, 51, 59, 60]. In the context of TSAs and GSAs, the chemical shifts of ^19^F resonances provide a key measure of interactions between MF_*x*_ moieties and their protein hosts, and report the electronic environment of the fluorine nuclei. When combined with NMR computations, they also act as indirect reporters of changes in electronic environment experienced by phosphoryl oxygen atoms in transfer reaction TSs [44, 61, 62]. ^19^F NMR resonances display a high degree of dispersion and are calculable with good precision from QM analysis of electronic distribution [37, 51, 63], showing resonances strongly affected by neighboring H-bond donors. Reduction in the number of H-bond partners generally results in upfield shift of ^19^F resonances, as shown clearly in a comparison of the G6P and the 2-deoxy G6P complexes of βPGM (−18.1 ppm) [53]. In general, the resonance of the fluorine coordinated to a catalytic magnesium is always the most upfield, because of depletion of H-bond coordination [44, 45, 50, 51, 53, 60, 62]. Proton distribution near fluorine nuclei can be further assessed through the quantitation of ^19^F-^1^H NOEs using perdeuterated enzyme in protonated buffer to suppress the ^1^H–^1^H spin diffusion [49, 51], while resonance assignment of exchangeable ^1^H nuclei in the protein enables unambiguous assignment of individual ^19^F NMR resonances. The number of H-bond donors can also be assigned based on solvent induced hydrogen/deuterium primary isotope shifts (SIIS) of ^19^F NMR resonances. For NH···F and OH···F H–bonds to MF_*x*_ moieties, the SIIS size reflects local proton densities [64], and this has been used to assign F_A_, F_B_, and F_C_ in a βPGM·MgF_3_^–^·G6P TSA complex [62].

Scalar coupling between nuclei involved with N–H···F H-bonds is an additional parameter that shows details of the coordination of the MF_*x*_ moiety by the protein. ^1^ *J*_HF_ and ^2^ *J*_NF_ couplings have been reported for individual NH···F pairs, with values up to 59 and 36 Hz, respectively [62]. All the effects described above, SIIS, NOE, chemical shifts, and scalar couplings, correlate closely with H-bonding orientations and distances obtained from high resolution crystal structure analysis. ^19^F chemical shifts are invariant over the pH range 6.5–9.5, they signal that there is no detectable change in protonation state of the enzyme in the environment of the TS complex, but the pH dependence of ^19^F NMR resonances and multiplicity can identify a switch from AlF_4_^–^ to MgF_3_^–^ complexes above pH 8, as illustrated for cAPK (Fig. 14c) [57].

NMR measurements of ^19^F nuclei in the active site of MF_*x*_ TSA complexes thus provide a picture of charge distribution between the phosphoryl group mimic and the protein. The good relationship between ^19^F NMR chemical shifts and SIIS values illustrates the dominant influence that very localized H-bonds have on shaping charge density on MF_*x*_ moieties.

## Computational Analyses of MF_*x*_ Complexes

### Balancing Accuracy of Energy/Structure and Conformational Sampling

A computational simulation of the structure and bonding of a biochemical system at atomic resolution has two demanding features:

1. The solution of accurate molecular energies, ideally with as little parameterization as possible;
2. The exhaustive consideration of relevant conformations of macromolecules.

For the simulation of biomolecules, it is unavoidable that both criteria must be approximated to varying degrees. In practice, different computational methods put different emphasis on one or the other of these two features. Any useful calculation must meet both criteria adequately. Solutions of the energy of a macromolecule, and thence its structure, should be made for each conformer of the molecule. Hence, the task of achieving reliable energies severely raises the cost of the computation. This constraint therefore drives down the number of conformers to be computed, with the risk that the program may fail to examine the specific conformation most relevant for the reaction under investigation.

To attain a compromise between these two features, the methodology used has to strike a balance between defining a central quantum mechanics (QM) zone and a molecular mechanics (MM) zone dealing with the major part of the macromolecule and environment. The combination of the two regions is called a QM/MM calculation. A QM description is necessary to describe bond–breaking–making processes or electronic excited states because molecular mechanics cannot describe these phenomena. Different balances between these two features are achieved by different choices in the apportionment of resource to the QM region. These include Kohn–Sham density functional theory (KS-DFT) [65–69] and empirical valence bond (EVB) [70, 71], while similar choices exist for the MM zone. However, the QM zone is the priority region.

### Tradeoff in Accuracy of Energy/Structure: Parameterization Simplification vs. Mathematical Complexity

Accurate molecular energies can be obtained in an unbiased, systematically correctable manner [72–74] to get the desired accuracy. However, the computational resource required is very expensive, and is usually unacceptable because resource must be apportioned to adequate conformational sampling. In general, either an approximate QM method such as KS-DFT is used, or a heavily parameterized model is designed for a specific system such as EVB. Briefly, parameterization can tailor a QM method specifically to that molecule under analysis—and thereby eliminate many mathematical degrees of freedom. Hence, the calculation can be performed rapidly and can incorporate greater conformational sampling, but it must rely on the assumption that the reduced mathematical form faithfully represents the true quantum mechanics. By contrast, the various KS-DFT forms have parameters which are fixed by the design of the functions, and are completely independent of that particular biomolecule under investigation. Thus, the application of KS-DFT to a specific biomolecule has no freedom to change parameters to suit the target. Hence, KS-DFT deploys a more general mathematical framework, and more faithfully echoes exact quantum mechanics within budget.

### Tradeoffs in Conformational Sampling: Dynamics vs. Statics

In order to balance the budget of the computation program, a choice has to be made between dynamics and statics. On the one hand, a *dynamics* description delivers an explicit femtosecond-by-femtosecond time evolution of the atoms, boosted by metadynamics [67]. On the other hand, a *statics* analysis of a few discrete critical points along the reaction identifies TSs and/or intermediates as maxima/minima along the reaction coordinate. Each has its strengths and weaknesses.

A dynamics computation shows the true time-evolution of the molecular system, especially how atoms re-arrange to move along all possible reaction paths, step-by-step. All possible chemical reactions/conformations are sampled in due frequency with the Boltzmann distribution of states. The computation does not “target” a specific reaction path. TSs are rare-events, require long simulations or metadynamics [67], and so demand a smaller QM zone to allow an adequately fast calculation. This reduction of the QM zone, relative to that for statics described below, makes possible the conformational sampling needed to find the right state. A balance has to be struck between faithfully computing dynamics or prioritizing accurate energy calculations.

The choice for statics in following a reaction path, selected a priori, enables easy identification of the TSs for bond-breaking-making using standard quantum chemistry algorithms. Mathematical properties of energy maxima (TSs) and minima (intermediates) can be sought automatically. Users can seek out any desired pathway, but they have to sacrifice an understanding of the relative values of each path. This requires minimal computational resource compared to that required for a dynamics calculation, and so can accept a much larger QM region and/or a more accurate QM calculation. However, the a priori choice of the conformation is risky: it depends strongly on the accuracy of choice of the *true* TS conformation, which may or may not be found among existing crystal structures in the PDB.

**Fig. 15:**
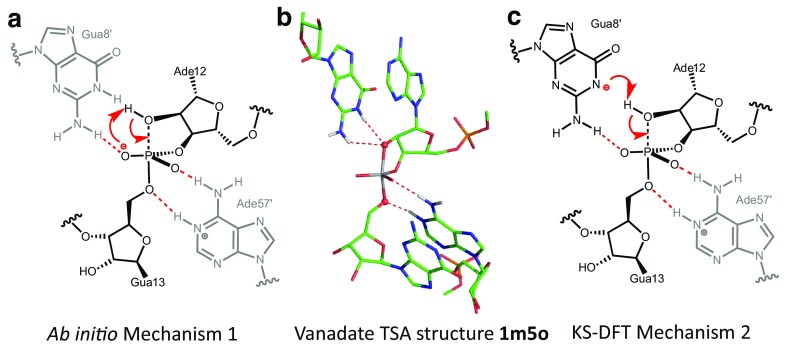
**a** Ab initio mechanism for the first step of the hammerhead ribozyme reaction showing PT from Ade12-O2’ to PO^2^ in the formation of a transient pentaoxyphosphorane species. **b** Crystal structure of the hammerhead ribozyme as a tbp vanadate complex (PDB: **1m5o**). **c** DFT computed mechanism for PT to the anionic Gua8’ preceding bond formation from P to Ade12-O2’ (colors: carbon, *green*; nitrogen, *blue*; oxygen, *red*; vanadium, *grey*; H-bonds, *red dashes*)

Take for example the first mechanistic step of the hairpin ribozyme. This is cleavage of the bond from the 3′-phosphate of A-12 to the 5′-oxygen of G13 to form a 2′,3′-cyclic phosphate which has been modeled as a pentacoordinate vanadate TSA structure (PDB: **1m5o**, 2.2-Å resolution; Fig. 15a). The two proximate nucleobases are G8’ and A57’ whose catalytic roles are controversial: there is good support for protonation of A57’-N^1^ but some debate whether G8’ is deprotonated on N^1^ or not. The computation accepted formation of an intermediate pentacoordinated phosphorane and then posed the question: “How and when is the proton removed from A8-O2’ and transferred to PO^2A^?” A thorough benchmark study of comparative QM/MM methods has been applied to this mechanism [75], comparing an ab initio method (i.e., no parameterization) with a KS-DFT method (a small number of fixed parameters). As the energy differences for the two paths should be comparable, a careful QM analysis was necessary. Ab initio energy barrier prediction matched experimental estimates, giving good support to Mechanism 1 (Fig. 15b) with a direct four-center proton transfer (PT), not involving a neutral G8’.^3^ However, it was observed that the flow of atoms predicted by the parameterized method was inconsistent with benchmark calculations. The authors therefore employed umbrella sampling in a KS-DFT analysis to achieve dynamics convergence, and found Mechanism 2 to be preferred with the anionic G8’ acting as a base to abstract the proton from Ado12-O2’ (Fig. 15c).

### KS-DFT as a QM Region Description

The most commonly chosen methodology for describing the QM portion is KS-DFT^4^ functionals. This makes a practical compromise between precision and cost of computation (Sect. 7.2). KS-DFT dispersion-corrected functionals can now describe molecular geometries to within 0.02 Å [76–78], and are particularly valuable because their description of energies and geometries is unbiased. They have been shown to describe basic bond-breaking behavior, H-bonding, and energetics [77–81]. In a QM/MM calculation, the boundary region at the interface between the QM zone and the MM zone can be problematic because the energies on the QM side need not be the same as those on the MM side. Any mismatch between kinetic and potential energies across the boundary leads to un-physical behavior. This boundary problem can be eliminated by depriving large portions of the macromolecule of an MM force field, which calls for a tradeoff between:

1. A more faithful representation of long-range chemical interactions and a potentially problematic boundary between zones introducing artifacts; and
2. Neglect of long-range chemical interactions altogether, with no un-physical artifacts introduced by the QM/MM boundary.

**Fig. 16:**
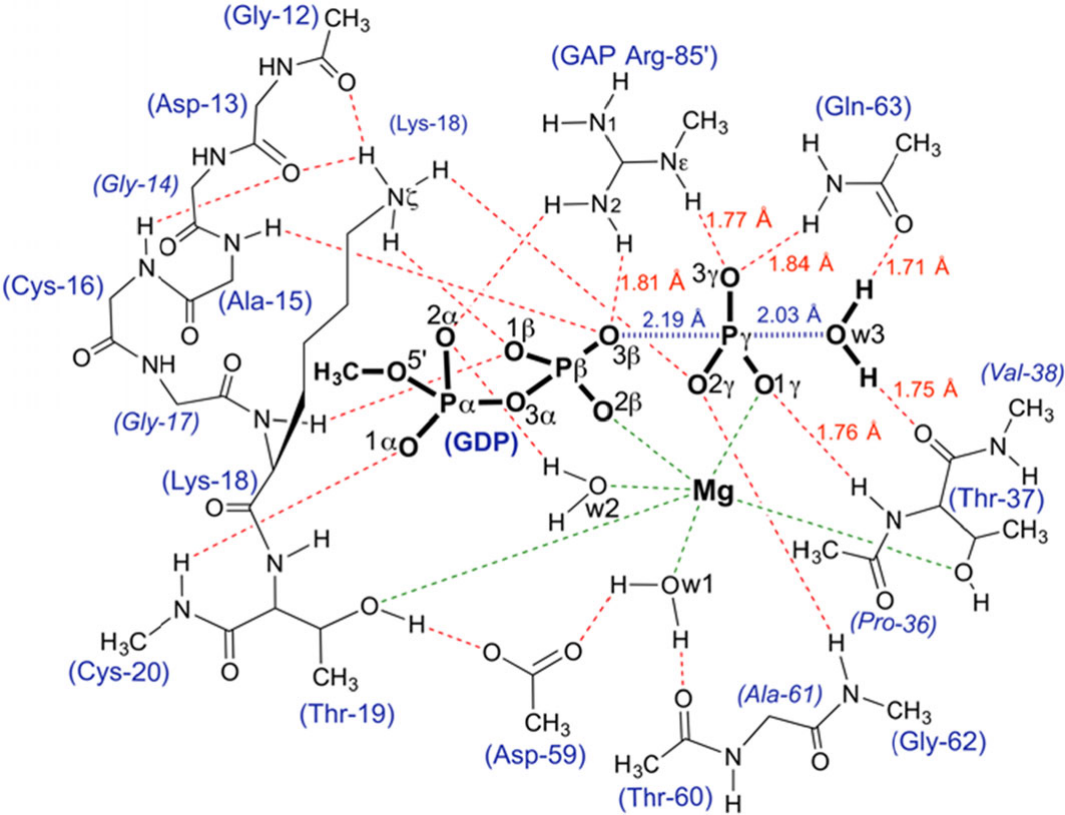
Atoms in the QM zone for KS-DFT computation of the TS for GTP hydrolysis by RhoA/RhoGAP showing the 21 H-bonds in the catalytic network (*red dashes*) with ligands coordinated to Mg (*green dashes*). Amino acid residues are numbered according to RhoA sequence plus Arg85’ from RhoGAP (Figure taken from Ref. [44])

Either choice is problematic, and a case-by-case decision must be made. In some cases, to avoid boundary complexities exclusively QM calculations have been used, usually KS-DFT. They usually rely wholly on experimental data from the structure of a TS mimic, thereby obviating the need for a conformational search, and allowing full investment of the computational resource to maximize the size of the QM zone. For example, a recent study of GTP hydrolysis by RhoA/GAP to identify the reaction mechanism employed a large QM calculation [44]. The KS-DFT zone was large enough to embrace the reacting methyl triphosphate, its coordinating magnesium and nucleophilic water, and also residues from some 18 additional amino acids that contribute to the stability of a network of 21 H-bonds which deliver the conformation of the TS for water attack on P^G^. Successive rounds of DFT computing established that contributions from atoms in the third solvation shell of the transferring phosphoryl group were required to deliver stability. The result was a QM region of 91 heavy atoms (181 total atoms) (Fig. 16). Because the starting TSA structure (PDB: **1ow3**) was of sufficiently high resolution (1.8 Å) to give confidence that the study was based on a reliable model of the TS, the addition of an MM contribution was bypassed, obviating the need for a QM/MM boundary. However, this limited the computational output to geometric and spectroscopic features. The absence of conformational sampling, sacrificed because of the large and very expensive QM region, also limits comment on activation energies.

The iterative computational procedure delivered a mechanism in which the nucleophilic water is doubly protonated with H-bonds to carbonyl oxygens of both T37 and Q63 residues until after the TS for bond making/breaking, thereby orientating the nucleophilic water for good orbital overlap with the antibonding O^3B^-P^G^ σ* orbital. PTs are not seen in the TS, but occur subsequently.

### EVB as a QM Region Description

The Empirical Valence Bond method deploys a simplified mathematical framework to achieve the most rigorous possible conformational sampling. In essence, the EVB framework is largely a molecular mechanics based method, with the exception of its representation of a single “orbital” for each molecule, identified as involved in the bond–breaking–making reaction. No other electrons/orbitals are represented explicitly. This framework thus imposes the presumption that only a single orbital is involved in the bond-reorganization for a reaction. The EVB parameterization process is fundamentally chemistry-imposed: it identifies, a priori, what orbitals are involved and dictates chemistry-based molecular mechanics energy functions. This is in sharp contrast to a KS-DFT prescription of a QM region, which is fundamentally agnostic of chemistry, not defining bonds or selecting orbitals targeted for reaction, but merely defining a total number of electrons and nuclei involved, with no presumption of chemistry. As a result of the EVB simplifications, larger-scale changes in molecular conformation can be observed. In this way, the initial conditions of the experimental crystal structure are not a trap; the computational protocol allows the biomolecule to move freely.

**Fig. 17:**
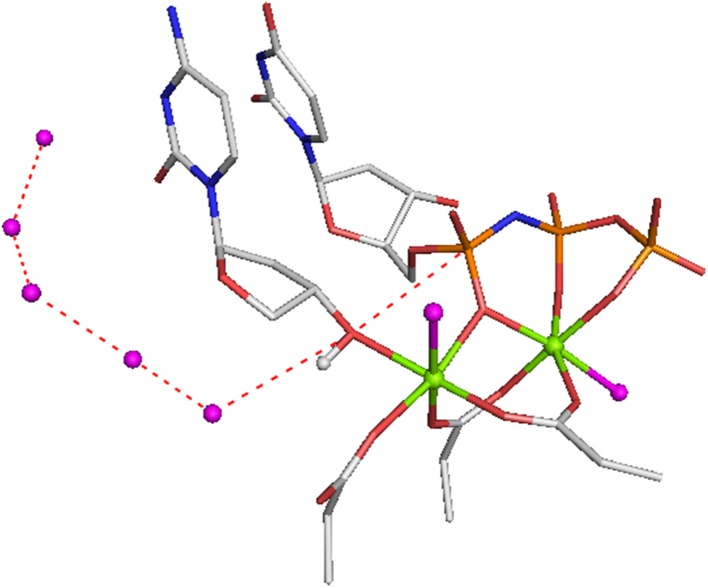
EVB analysis of DNApolβ showing atoms used in the QM region, augmented by additional water oxygens (*magenta*) and the focal hydrogen undergoing transfer (*white sphere*), and taken from PDB: **4fms**

A study of the mechanism of DNA polymerase β provides a good example of the application of the EVB methodology [82]. The questions under examination were (i) the destination and timing of PT from the nucleophilic 3′-OH, with three aspartates and water as potential acceptors, and (ii) the concerted or stepwise nature of phosphorus migration.^5^ The starting structure was native DNApolβ (PDB: **2fms**, 2.0 Å resolution) and some 70 heavy atoms were included in the QM zone (Fig. 17), linked to the assumption that the reaction takes place in the three steps: a PT from the primer 3’OH, followed by in-line nucleophilic attack of 3’O^–^ on the dUTP α-phosphate, with reaction completed by departure of pyrophosphate. Particular attention was paid to the electrostatic role of the two catalytic magnesium ions through the course of the reaction. The computations were guided by consideration of the pH-rate profile for wt and mutant polymerases and focused on seven EVB states. This led to the conclusion that PT from the nucleophilic hydroxyl group is to bulk water via a chain of water molecules that extends into the active site, rather than to any of the three neighboring aspartates (as favored in previous studies) and precedes P–O bond formation. The phosphorylation process was found to be associative with a pentacovalent intermediate,^6^ though the investigation warned that extensive sampling is essential for EVB analysis of reaction mechanisms.

### The Use of MF_*x*_ in Computational Studies of Enzyme Mechanisms

**Fig. 18:**
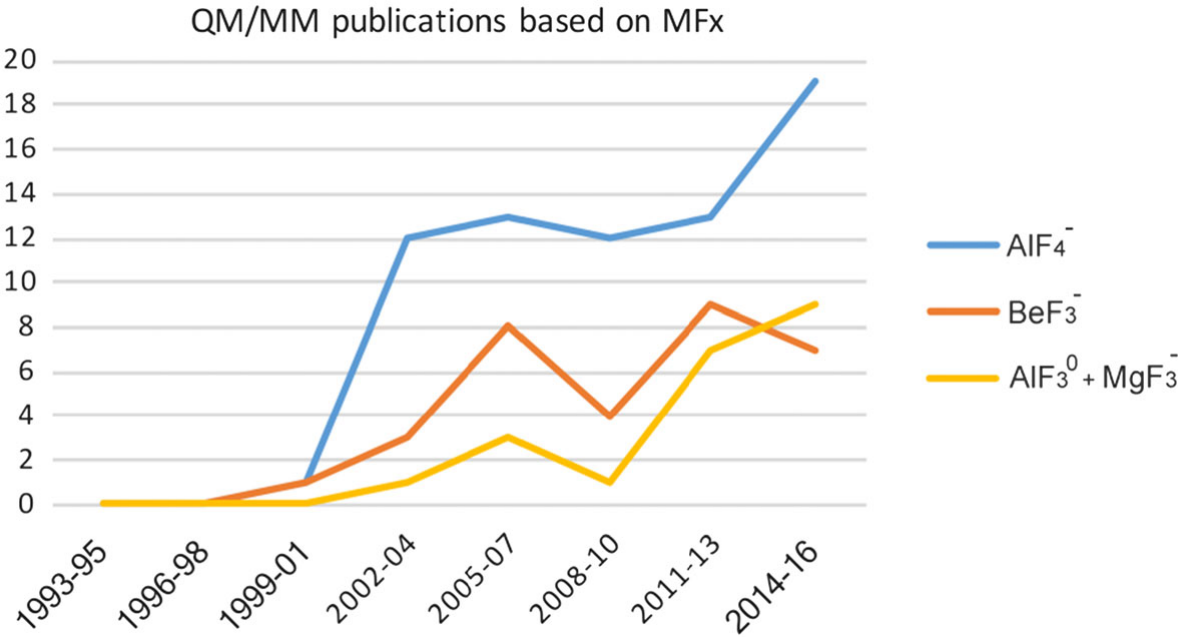
Growth of computational publications since 1994 showing QM results on AlF_4_^–^ leading with [AlF_3_^0^ + MgF_3_^–^] comparable to studies based on BeF_3_^−^ complexes

As has been described above, protein trifluoroberyllate complexes are fundamentally different from those involving metal fluorides of aluminum and magnesium. They have closely similar tetrahedral geometry and net charge to the parent phosphate, their macromolecular structures have folds and atomic organization that relate to the ground state structures they mimic. On the other hand, AlF_4_^–^, “AlF_3_^0^”, and MgF_3_^–^ complexes have geometries that bring two axial ligands into alignment and proximity typical of the TS for concerted phosphoryl transfer, and this results in protein folds and atom coordination that resembles the TS for reaction. Computational enterprises have taken up both of these opportunities for a plethora of purposes. The growth in such studies is illustrated in the chart (Fig. 18). In practice, the three MF_*x*_ categories converge for the majority of computer purposes as they are usually transposed into PO_3_^–^ at an early stage in the computation. Therefore, the major part of the following description of computational studies on mechanisms of phosphoryl transfer will be focused on the target protein.

#### Validation of MF_*x*_ as a TSA for Phosphoryl Transfer

Relatively few computational studies have been directed at the structural identity of the MF_*x*_ complex per se. A contentious 1.8 Å resolution structure (PDB: **1o03**) focused on a six-atom tbp complex for βPGM, initially described as a pentaoxy-phosphorane [84]. They have converged on identification of (a) the observed crystal structure as a five-coordinate trifluoromagnesate complex rather than a five-coordinate phosphorus [24], (b) an active site stabilized by an extensive H-bonding network, and (c) a concerted transfer of the phosphoryl group without a stable phosphorane or metaphosphate intermediate [85–87]. They concluded that MgF_3_^–^ is a good TSA that can give insight into the geometry of the phosphoryl transfer TSs. A second example is a QM/MM analysis of the atomic nature of an MF_*x*_ moiety in a TSA complex for the key kinase, cAPK [61]. The structure of a tbp complex for cAPK·ADP·MF_*x*_ was originally described as AlF_3_^0^ (PDB: **1l3r**) but QM/MM simulations suggest that MgF_3_^–^ is the correct description of the tbp moiety rather than AlF_3_^0^, and that MgF_3_^–^ is a near isosteric fit to PO_3_^–^ in the computed TS for the hydrolysis of ATP [61, 88]. This result agrees with a ^19^F NMR analysis, have been directed at MF_*x*_ complexes for cAPK [50]. The computations conclude that this kinase prefers a monoanionic analog (MgF_3_^−^ or AlF_4_^−^) over a neutral analog (AlF_3_^0^) to match the −ve charge on the phosphoryl group.

#### Studies Linking Reaction Mechanisms from Model Systems to MF_*x*_ Enzyme Complexes

Early QM studies on phosphoryl transfer analyzed the hydrolysis of methyl phosphate [89] and methyl pyrophosphate [90], added magnesium [91], and then transposed the results into the context of the Ras GTPase active site. The result does not match well to the MF_x_ structure for Ras·RasGAP (PDB: **1wq1**) because (i) the computed O_A_—O_D_ separation lies in the region 4.7–5.5 Å and in the MF_*x*_ structure is 4.4 Å. (ii) The computation calls for a second water to facilitate PT [92], however, in those (few) instances where a second water is seen in high-resolution MF_*x*_ structures for Ras, it occupies the site vacated by a displaced or missing Gln61 residue, and is in no position to deliver the proposed catalysis (Fig. 7a).

### Computations Transposing GDP·MF_*x*_ into GTP Enzyme Complexes

#### Ras Family and GTP Hydrolysis

The use of MF_*x*_ TSA structures to identify the TS for hydrolysis of GTP by Ras proteins has been the basis of many computations. Several studies have used PDB: **1wq1** [22], the 2.5 Å-resolution structure of Ras·RasGAP·GDP·AlF_3_^0^ as starting point, and have employed both QM/MM [92–99] and EVB approaches [100, 101]. Some of these have aroused expert criticism of limitations inherent in the QM/MM approach [102]. The results have varied widely, from a two-step reaction mechanism with bond breaking preceding bond making (i.e. a dissociative process; Scheme 1a) [100], to exclusion of water by the arginine finger [98], tautomeric catalysis [17], electrostatic catalysis [101], a two-water mechanism [92], and sundry rationalizations of the adverse effects of mutations [97, 99, 101]. The QM zone has generally been limited to 30–40 heavy atoms and, in consequence, has not examined the role of the function of several amino acids in contact with the reactants, most especially the extensive H-bonding network (as in Fig. 16). By contrast, an alternative computational approach using Kohn–Sham DFT analysis for RhoA·RhoGAP hydrolysis of GTP employed a QM zone of 91 heavy atoms, embracing a network of 21 H-bonds, and has attributed catalysis to orbital orientation determined by protein control of H-bonds donated by the nucleophilic water (Fig. 16) [44]. The same study validated the high relevance of MgF_3_^–^ as a TSA by back-computing its structure from that of the calculated structure for the true TS complex for GTP hydrolysis.

#### Other GTPases and GTP Hydrolysis

A study on the structures of a GMP·AlF_3_^0^ complex (PDB: **2b8w**) and a GDP·AlF_4_^−^ complex (PDB: **2b92**) for hGBP1, has linked a mechanism for the hydrolysis of methyl triphosphate (MTP) to the two-step hydrolysis of GTP to GDP and thence to GMP by this interferon-activated human GTPase (Fig. 7b). The computation employed dated ab initio QM/MM molecular dynamics to simulate the hydrolysis of both GTP and of MTP as a reference system [103]. The study proposes that GTP hydrolysis involves an indirect, substrate-assisted catalysis mechanism, identifying the nearest general base as Glu99, which is 6.2 Å from the nucleophilic water in the TSA complex. This separation problem was resolved by invoking transmission of base catalysis via one water to Ser73, and thence via a second water to the nucleophilic water. These bridging waters are not present in the substantive (3.2 Å resolution) TSA complex but appear to be imported from a structure of hGBP1 with β, γ-imino-GTP that is clearly an NAC complex (PDB: **2bc9**; 2.8 Å resolution). This investigation merits a cautionary comment on the frailties of a computational analysis based on structures of poor resolution, under-informed by an adequate grasp of mechanisms of phosphoryl transfer.

### Computations Transposing ADP·MF_*x*_ into ATP Enzyme Complexes

#### ATP Hydrolysis by Myosin

**Fig. 19:**
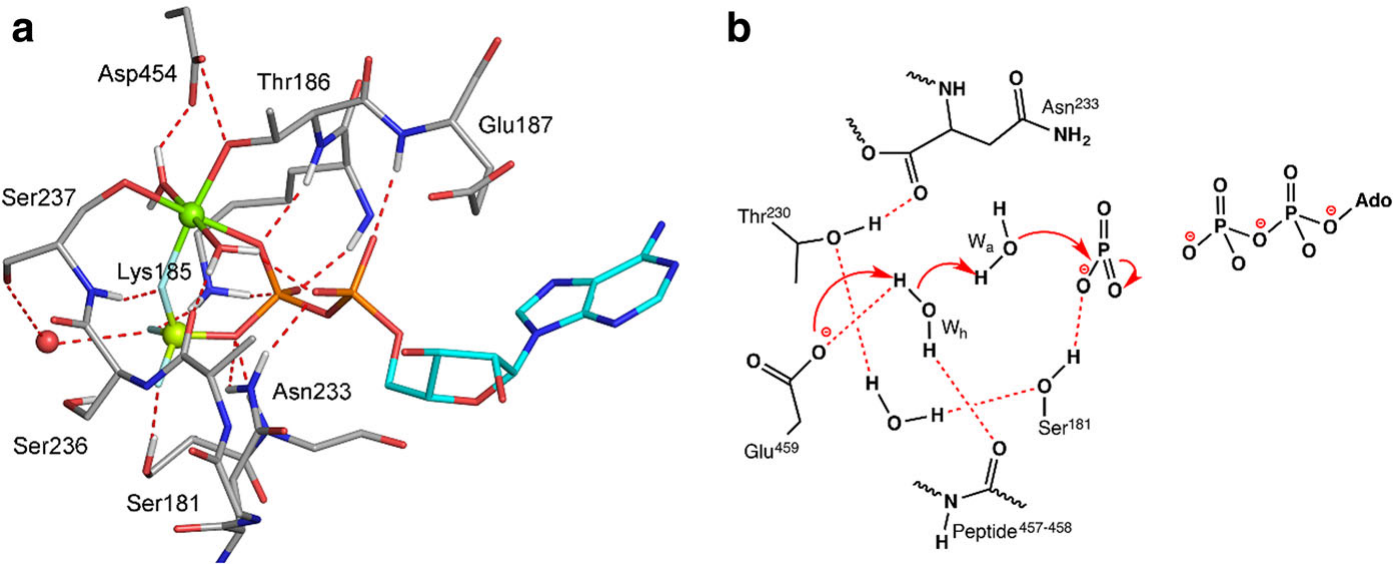
Mechanism of ATP hydrolysis by myosin. **a** Myosin complex with Mg·ADP·BeF_3_^−^ (PDB: **1mmd**) at 1.95 Å, with beryllium (*lemon sphere*) trifluoride bonded to O^2B^ of ADP (*cyan*). Amino acids in network (*silver, blue, red*) shown with H-bonds to TSA complex (*red dashes*). Nucleophilic water (*red sphere*) is in a NAC. **b** The key feature of the metaphosphate state is the extreme polarization of water *W*_a_, due to two H-bonds with the Ser237–C=O and water. Attack of *W*_a_ on P^G^ involves attendant PTs via the helper water, *W*_h_ (*red arrows*) [104]

Myosins are a family of ATP-dependent motor proteins whose role in muscle contraction is driven by ATP hydrolysis. Multiple structures of Mg·ADP·MF_*x*_ exist, including BeF_3_^–^ (PDB: **1w9i** and **1mmd**) and AlF_4_^–^ (PDB: **1w9l** and **1wj9**). These structures have been used to identify the catalytic amino acids and locate key water molecules, especially the nucleophilic water that attacks in-line at P^G^ (Fig. 8a). A recent QM/MM computation of the hydrolysis of ATP used a DFT method with B3LYP functional and a 6–31G(d,p) basis set to treat 84 atoms in the active site for the Mg·ADP·BeF_3_^–^ structure of the myosin II head group (PDB: **1mmd**), with ATP modeled by replacing BeF_3_ with a phosphate (cf. Fig. 4a) [104]. Although the starting structure for the computation (Fig. 19a) has the nucleophilic water (*W*_a_) in a NAC, as defined by its H-bond proximity to *F* ^2^ (2.7 Å) and out-of-line angle for the attack on Be (152˚), the simulations delivered a H-bond network that lead to the final product, H_2_P^G^O_4_^−^. The proposed mechanism involves formation of a stable metaphosphate intermediate prior to the TS followed by a series of PTs (Fig. 19b).

#### ATP Hydrolysis by F1 ATPase

**Fig. 20:**
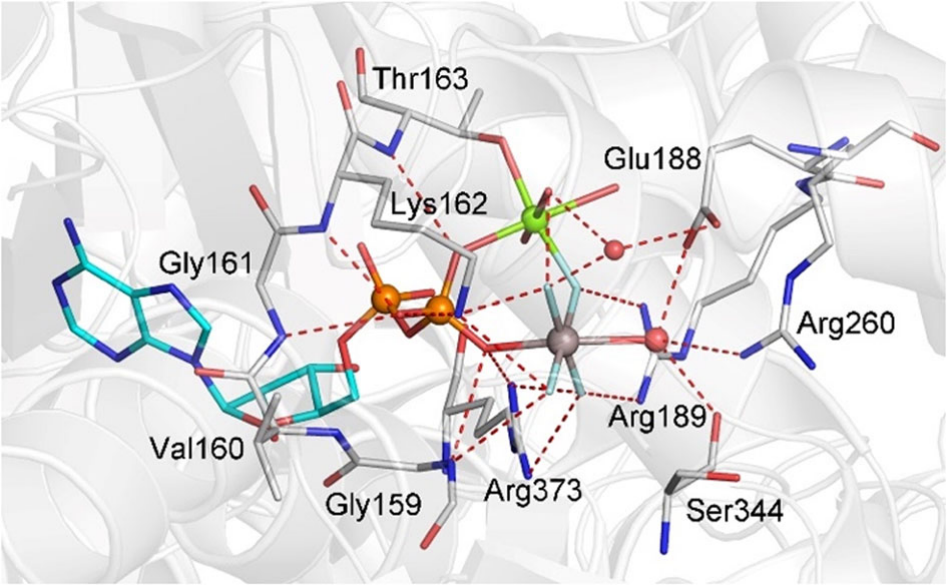
F1ATPase·Mg·ADP·AlF_4_^−^ TSA complex for ATP hydrolysis (PDB: **1h8e**) showing in-line water attack on aluminum (*red sphere*) (color: phosphorus, *orange*; amino acids, *gray*; adenosine, *cyan*; waters, *red sphere*)

F1-ATPase (ATP synthase) is a membrane-bound protein that uses a proton gradient to drive ATP synthesis. There are three prime MF_*x*_ complexes for the α_3_β_3_ assembly at resolutions from ≥2.0 Å (PDB: **1w0j**, **1h8e**, and **1e1r**). These have been starting points for multiple computational studies, of which the majority are concerned with energetics of the chemical step, coupling between the subunits and the rotor, and rotational behavior of the synthetic complex [102, 105–107]. More recent studies involve the juxtaposition of several structures from the PDB. One of these builds a visual comparative structural approach that emphasizes pivotal roles for Mg^2+^ and protein P-loop residues in synthesizing ATP (159–163). It uses four structures, including the ATPase·Mg·ADP·AlF_4_^–^ complex (PDB: **1h8e**; Fig. 20) [108].

#### Phosphoryl Transfer in Kinases

The catalytic subunit of cAPK is a serine/threonine kinase responsible for many of the effects of cAMP signaling. It is a prototype for the kinase family that uses two catalytic magnesiums, and has become the most widely studied of all kinases. Many computations have focused on the phosphorylation of a serine in the target peptide by ATP, but recent advances in high-resolution structures of an NAC complex with β,γ-imino-ATP and the products from its slow reaction during crystallization combined with an MgADP·AlF_3_^0^ TSA, (PDB: **1rl0**) [109] have given new opportunities for computational analysis. One of these, using MP2/aug-cc-pVTZ/CHARMM//B3LYP/6-31 + G(d)/CHARMM electronic structure calculations with a completely solvated model of the cAPK_cat_–ATPMg_2_–SP20 system finds that a dissociative concerted mechanism involving two consecutive steps is more favorable than an associative mechanism [110, 111] or a concerted loose mechanism [112] (Scheme 1b). In step 1, phosphoryl transfer involves a dissociative TS with an O–P^G^–O distance of 4.7 Å. Then, step 2 follows with back-protonation of the serine phosphate.

**Fig. 21:**
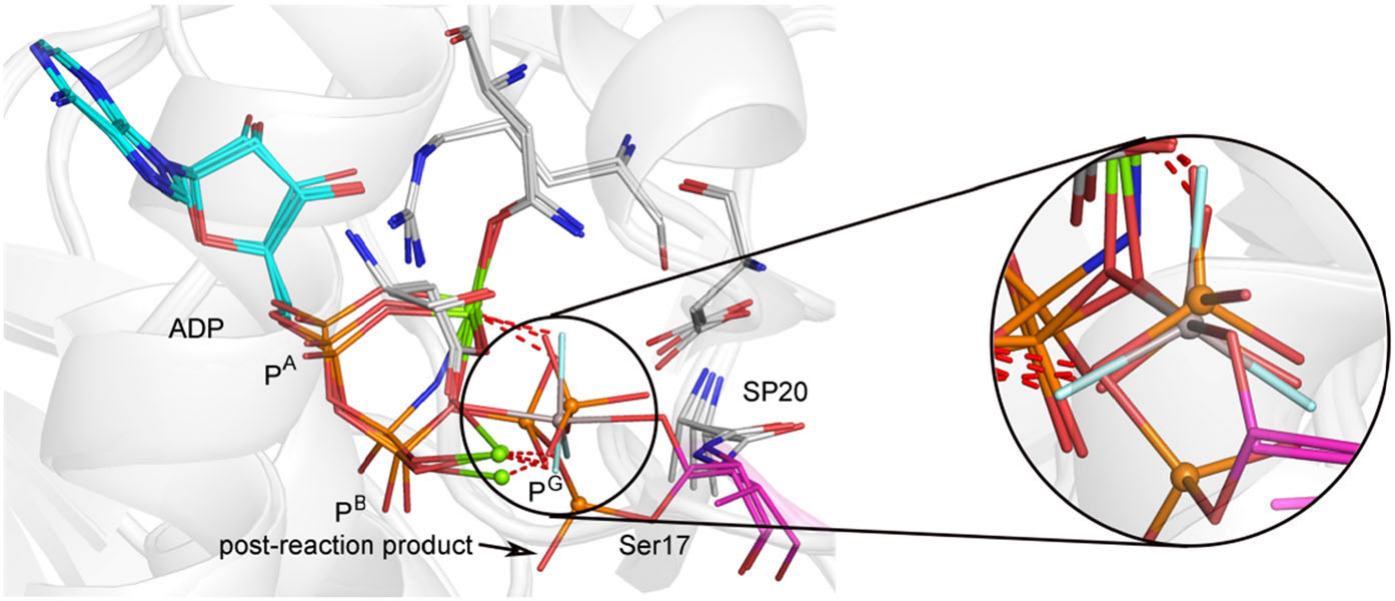
Superposition of the active site of cAPK for 55% reactant and 45% product (PDB: **1rdq**, *red sticks*), TSA (PDB**: 1r3l**, *gray sticks*), and product (PDB: **4hpt**) after conformational change post-reaction. Alignment of invariant amino acids (*silver*) and adenosine residues shows the high quality of fit (aligned for all Cα). The *inset* shows an orthogonal view of the threefold overlay of P^G^O_3_ and its mimic

The range of analyses to be found in such computations has to be set against recent structural work. The superposition of well-resolved complexes of cAPK with reactant, MgADP·MgF_3_^–^ TSA analog (PDB: **1l3r**), and a product complex (PDB:**1rdq**) [113] shows structurally that the overall reaction is defined by the geometry of active site residues and involves migration of phosphorus only 1.1 Å from start to finish with O_A_–O_D_ separation ~4.5 Å in the TS (Fig. 21). Other kindred analyses have shown similar results [26].

### Thoughts from Computations

The first phase of computational studies for phosphoryl transfer was largely focused on finding a close match between computed activation energies and the experimental ones. However, recent advances in computational methodology have cast a shadow on earlier methods, where the energy error might easily lie in the range of 2–10 kcal mol^−1^, with error spreading as large as 30 kcal mol^−1^ [78, 114, 115]. Current protocols enable a much larger number of heavy atoms to be embraced in the QM zone, leading to computer results that hinge on geometry of the TS and the H-bond network that it embraces [44, 108, 111]. Such analyses have identified the propensity of nucleotide analogs, particularly β,γ-iminoATP, β,γ-methyleneATP, and their GTP counterparts, to deliver NACs for phosphoryl transfer processes, which is now recognized in recent computational studies as capable of generating small but highly significant conformational changes in kinases and GTPases [44, 108]. Lastly, the belief that enzymes work by optimizing reaction mechanisms that work slowly in solution, as Knowles put it “Not different, Just better” [116], is proving to be wide of the mark for phosphate reactions. There is growing evidence that phosphoryl transfer takes place in a desolvated environment to enable full protein control of the catalytic region. Water is rigorously excluded to avoid disruption of H-bond networks that are *essential* for the organization of catalysis.

## Conclusions

Trifluoroberyllate, tetrafluoroaluminate, and trifluoromagnesate are the primary anionic MF_*x*_ species that can mimic the phosphoryl group. Structural, spectroscopic, and computational methods have combined to validate their use as surrogates for PO_3_^–^ in ground state and transition state analog complexes for many enzymes. Their use has delivered details of phosphoryl transfer at atomic resolution and supported investigations of protein folding and aggregation for tertiary structure problems. In particular, their analysis has confirmed existing concepts, introduced new ideas, and set new goals, of which the following comprise a brief summary:

- In-line stereochemistry and concertedness for S_*N*_2(P) reactions has been established at atomic resolution;
- Relative priority of charge over geometry in transition state organization is well supported;
- Subtle conformational differences between NAC and TS conformations of amino acid functions are increasingly apparent;
- The role of H-bond networks to give structural coherence to proteins in transition states is burgeoning;
- The propensity of anionic phosphate oxygens to H-bond to ROH nucleophiles explains the need for solvent exclusion from the TS, while its impedance offers a new interpretation of general base catalysis.

## Footnotes

1 Lord Todd of Trumpington, Nobel Laureate 1957, 1702 Professor of Chemistry 1944-71, Cambridge, UK.

2 Professor of Chemistry, 1953-2007, Harvard, Cambridge, USA.

3
*NB* In other computational studies, such four-center PTs for phosphoryl reactions have been deemed to be very high energy.

4 In literature meant for using DFT in organic, biological, or inorganic applications, “KS-DFT” and “DFT” are used largely interchangeably. Theoreticians draw a distinction between these terms; KS-DFT is a subset of DFT for the given selection of expressing the kinetic energy in terms of orbitals.

5 A previous study favored a concerted reaction path (Lin et al. [83]).

6 It should be added that recent structural studies suggest that one of the catalytic magnesiums is lost from its pre-TS location during the bond-making-breaking process and then appears in a new location (PDB: **4klf** and **4klg**).
